# Spatiotemporal evolution of aroma compounds in egg white and yolk of preserved eggs during pickling

**DOI:** 10.1016/j.fochx.2026.104483

**Published:** 2026-09-19

**Authors:** Xiaoqian Chen, Ziyan Yue, Yifei Li, Weixi Yang, Shugang Li, Xiaobin Ma, Maurice O'Sullivan, Hong Zeng, Yanbo Wang

**Affiliations:** a Key Laboratory of Geriatric Nutrition and Health, Ministry of Education, School of Food and Health, Beijing Technology and Business University, Beijing 100048, China; b School of Food and Health, Beijing Technology and Business University, Haidian, 100048 Beijing, PR China; c Key Laboratory for Animal Food Green Manufacturing and Resource Mining of Anhui Province / Engineering Research Center of Bio-process, Ministry of Education / School of Food and Biological Engineering, Hefei University of Technology, Hefei 230601, China; d School of Food and Nutritional Sciences, University College Cork, T12 Y337, Cork, Ireland

**Keywords:** Preserved eggs, Aroma evolution, Metabolomics, Volatile compounds

## Abstract

Preserved eggs (PEs) are a traditional Chinese pickled product known for the distinctive flavor. However, the formation mechanisms of such iconic flavor remain unclear. In this study, electronic nose (*E*-nose), gas chromatography-ion mobility spectrometry (GC-IMS), gas chromatography–mass spectrometry (GC–MS), and metabolomics analysis were performed to investigate the spatiotemporal flavor evolution of preserved eggs during pickling. Temporal patterns were observed in pH changes, protein degradation and oxidation as well as lipid oxidation. A total of 3 key aroma compounds were identified in egg white while 23 were identified in egg yolk, among which 1-octen-3-ol contributed most to the overall flavor. Lipids and amino acids may serve as the main precursors for the formation of key aroma compounds. This is the first study that reveals the spatiotemporal evolution of key aroma compounds in the egg white and yolk of preserved eggs, laying a solid flavor chemistry foundation for targeted flavor control.

## Highlights

• Aroma evolution of egg white and yolk during pickling was investigated separately.
• The early to mid-stage of pickling was identified as the critical period for flavor formation.
• 1-Octen-3-ol was the dominant aroma contributor in preserved eggs.
• Lipids and amino acids may serve as the primary precursors for flavor compounds.

## 1 Introduction

Preserved eggs (PEs, also known as century eggs or Pidan) are a traditional Chinese pickled egg product with a long history of two thousand years (Zhang et al., 2018). China is one of the largest producers of duck eggs, with approximately 40% of fresh duck eggs utilized for the production of PEs (Wang et al., 2021). In modern industrial production, PEs are manufactured using fresh duck eggs as raw materials, which are then cured in pickling solutions containing alkali, metal ions, and sodium chloride (Wang et al., 2021). PEs are nutritionally rich, and their unique high-alkaline pickling process induces protein denaturation and degradation into small peptides and amino acids, thereby enhancing nutrient absorption (Jia et al., 2025). In addition, PEs exhibit high biological activity, including anti-inflammatory effects and modulation of gut microbiota, making them a functional food suitable for consumers of all ages (Batool et al., 2021, Li et al., 2023). Meanwhile, the unique flavor and texture of PEs have made them widely popular among consumers in China and Southeast Asia (Chen et al., 2025).

Currently, research on PEs has primarily focused on their protein gel properties and flavor characteristics, particularly the volatile compounds in egg yolk. For example, Zhang et al. (2018) characterized the potential aroma compounds in preserved egg yolk using gas chromatography-olfactometry (GC-O). The characteristic flavor of PEs is largely attributed to the degradation and oxidation of proteins and lipids under alkaline conditions during pickling (Xue et al., 2022). However, studies on the formation pathways of these key aroma compounds in egg yolk remain limited, and the formation and evolution of volatile compounds in preserved egg white during pickling are still unexplored. Furthermore, most studies have focused on the flavor at the end of pickling, with few investigations addressing the spatiotemporal evolution of flavor across the entire pickling process, including differences between egg white and yolk.

With the development of analytical instrumentation, GC–MS and GC-IMS have become important techniques for food flavor analysis. GC–MS possesses strong separation and identification capabilities but shows limited sensitivity for trace volatile compounds (Chen et al., 2021). In contrast, GC-IMS offers rapid detection and high sensitivity, but its database remains incomplete (Parastar & Weller, 2024; Xiao et al., 2022). Therefore, the integration of GC-IMS and GC–MS provides a more comprehensive characterization of volatile compounds in food samples (Zhao et al., 2025). In addition, metabolomic analysis enables the identification of small-molecule metabolites, providing insights into the composition and transformation of non-volatile compounds during processing, which play a key role in flavor development (Ruan et al., 2026). For instance, Cai et al. (2026) explored the flavor formation mechanism of Taihu goose egg white during low-temperature storage, and metabolomics analysis revealed the key role of amino acid in flavor formation.

This study used multi-omics techniques to reveal the spatiotemporal evolution of volatile and non-volatile compounds during pickling and to explore their relationship. GC–MS and GC-IMS identified volatile compounds, while untargeted metabolomics analyzed non-volatile compounds. Correlation network analysis was then used to explore the potential mechanisms associated with key aroma compound formation. These findings provide an essential flavor chemistry basis for quality control and flavor optimization strategies for PEs.

## 2 Materials and methods

### 2.1 Materials

Fresh duck eggs were purchased from Anhui Xianggongguan Ecological Agriculture Co., Ltd. (Anhui, China). Copper sulfate (CuSO4) was supplied by Jiangsu Kelunduo Food Ingredients Co., Ltd. (Jiangsu, China), sodium hydroxide (NaOH) was purchased from Binhua Group Co., Ltd. (Shandong, China), and sodium chloride (NaCl) was obtained from China Salt Jingjinji Salt Industry Co., Ltd. (Beijing, China). All other experimental reagents were of analytical grade.

### 2.2 Preparation of preserved eggs

Cleaned duck eggs were immersed in a pickling solution consisting of NaOH (4.5%, m/v), NaCl (4.0%, m/v), and CuSO_4_ (0.4%, m/v) at 25 °C for 36 days. The samples were collected at 0, 6, 12, 18, 24, 30, and 36 days. Egg white and yolk were carefully separated, then put into a refrigerator at −80 °C for further determination and analysis.

### 2.3 Physicochemical properties analysis

The pH of egg white and egg yolk samples at different pickling times was measured with slight modifications according to Luo et al. (2024). After homogenization, the pH was determined using a pH meter (FE28-Standard, Mettler Toledo, Zurich, Switzerland). The protein concentrations of egg white and egg yolk were determined using a BCA protein assay kit according to the method reported by Qi et al. (2024). The protein carbonyl contents of egg white and egg yolk were determined using the DNPH reaction method (Chen et al., 2025). Lipid oxidation was assessed by the thiobarbituric acid reactive substances (TBARS) assay. A slightly adapted method based on Chen et al. (2025) was used to determine TBARS values in egg yolk.

### 2.4 *E*-nose analysis

Characterization of the volatile flavor profiles of preserved egg samples was performed using a PEN3.5 E-nose (Airsense Analytics, Germany) equipped with ten metal oxide semiconductor sensors. The corresponding sensitive substances of each sensor are listed in **Table S1**. The method was adapted from Gao et al. (2023) with minor modifications. Briefly, 2 g of preserved egg white or yolk was placed in a 20 mL sealed headspace vial and incubated at 60 °C for 30 min to allow volatile compounds to equilibrate. The collected headspace gas was analyzed by the electronic nose system with a carrier gas flow rate of 500 mL/min and a measurement duration of 120 s. Between measurements, the sensors were purged with ultrapure air for 120 s to reestablish the baseline.

### 2.5 GC-IMS analysis

Volatile compounds in preserved egg samples were analyzed using a Flavor Spec® GC-IMS system (Shandong Haineng Scientific Instruments Co., Ltd., China) containing an MXT-5 capillary column (15 m × 0.53 mm, 1 μm film thickness). The method was adapted from Xiang et al. (2023) and Sun et al. (2026). Briefly, 2 g of preserved egg sample was added to a 20 mL sealed headspace vial, mixed with 15% aspartic acid and 2 mL distilled water, and incubated at 60 °C for 10 min to promote the release of volatile compounds into the headspace. The headspace gas (1 mL) was then injected into the GC-IMS system via an autosampler needle at 85 °C in a splitless mode. Nitrogen was used as the carrier gas, and the flow program was as follows: initial flow of 2 mL/min held for 2 min, ramped to 10 mL/min over the next 8 min, increased to 100 mL/min during the subsequent 10 min, and finally raised to 150 mL/min over the final 10 min. The drift gas flow was maintained at 150 mL/min. Retention indices (RIs) were calibrated using a standard mixture of n-ketones (2-butanone, 2-pentanone, 2-hexanone, 2-heptanone, 2-octanone, and 2-nonanone) under identical chromatographic conditions. The detected target compounds were comprehensively matched against the GC retention index database and IMS drift time database embedded in VOCal software, and qualitative identification of target analytes was performed based on the final matching outcomes.

### 2.6 GC–MS analysis

GC–MS analysis was performed according to the method described by Sun et al. (2026) and Yu et al. (2023), with slight modifications. Egg white and egg yolk samples collected at different pickling stages were analyzed separately. Briefly, 2 g of each sample was transferred into a 20 mL glass headspace vial sealed with a silicone septum. Subsequently, 3 mL of saturated NaCl solution and 10 μL of 2-methyl-3-heptanone (40.8 ng/g) were added as an internal standard.

The vial was equilibrated at 65 °C for 15 min to facilitate the release of volatile compounds. A headspace solid-phase microextraction (HS-SPME) arrow (120 μm, DVB/Carbon WR/PDMS, Shimadzu, Nakagyo-ku, Kyoto, Japan) was then exposed to the headspace under the same conditions for 25 min to extract volatile compounds. After extraction, the fiber was immediately inserted into the GC injector and thermally desorbed at 250 °C for 5 min for GC–MS analysis. GC–MS analysis was performed using a Shimadzu SH-PolarWax capillary column (60 m × 0.25 mm i.d. × 0.25 μm film thickness; crossbond polyethylene glycol stationary phase; Shimadzu, Kyoto, Japan). Helium was used as the carrier gas at a constant flow rate of 1 mL/min. The oven temperature was held at 40 °C for 3 min, ramped to 240 °C at 4 °C/min, and maintained for 10 min. Mass spectra were recorded in electron ionization (EI) mode at 70 eV, with the ion source temperature set at 230 °C, and full-scan acquisition was performed over a mass range of *m*/*z* 35–450. Volatile compounds were identified by comparing their mass spectra and RIs with the reference data available in the NIST library. The content of volatile compounds was calculated using the internal standard method (Sun et al., 2026). The equation for the calculation was as follows:

(1) $$C_{x}=\frac{S_{x}\times C_{0}}{S_{0}\times m_{1}}$$

where Cx is the semi-quantitative concentration of the volatile compounds, ng/g; Sx is the peak area of the volatile compounds; C_0_ is the concentration of the internal standard, ng/g; S_0_ is the peak area of the internal standard; m_1_ is the mass of the samples, g.

### 2.7 Sensory evaluation

Sensory evaluation was performed by a trained panel of 10 members (6 males and 4 females, aged 20–26 years). Panelists evaluated the odor attributes of preserved egg white and yolk samples at different pickling stages. Six odor attributes (fishy, ammonia, fatty, fresh, rotten egg-like, and salted egg-like) were scored on a 0–10 point scale, with higher scores indicating greater intensity of the corresponding odor attribute. Each attribute was defined using corresponding reference standards (**Table S2**). All panelists provided informed consent, and the study was approved by the Scientific Research Ethics Committee of Beijing Technology and Business University (approval No. 2026–110).

### 2.8 LC-MS analysis

Metabolites in preserved duck egg white and yolk samples were extracted using a modified protocol described by Wang et al. (2024). Approximately 1 g of each sample was combined with 20 mL of 70% methanol, vortexed thoroughly, and subjected to ultrasonic treatment in an ice-water bath for 10 min. The mixture was then incubated at low temperature for 15 min. After centrifugation at 12,000 rpm for 10 min at 4 °C, the supernatant was collected and stored at −20 °C overnight. Prior to Liquid Chromatography-Mass Spectrometry (LC-MS) analysis, samples were filtered through a 0.22 μm membrane. A pooled quality control (QC) sample was prepared by mixing equal volumes of all samples and was analyzed after every six sample injections to monitor the stability and reproducibility of the LC-MS analysis.

Chromatographic separation was performed on an Agilent ZORBAX Eclipse Plus C18 column (4.6 mm × 100 mm, 1.8 μm) using an ExionLC™ system coupled with a SCIEX X500R QTOF mass spectrometer (AB SCIEX). The column temperature was maintained at 40 °C with a flow rate of 0.4 mL/min, and the injection volume was 2 μL. Mobile phase A was water with 0.1% formic acid, and mobile phase B was acetonitrile with 0.1% formic acid. The gradient elution program was set as follows: 0–1 min, 95% A; 1–10 min, linear decrease of A from 95% to 50%; 10–12 min, 50% A; 12–18 min, linear decrease of A from 50% to 10%; 18–20 min, 10% A; 20–20.1 min, linear increase of A from 10% to 95%; 20.1–25 min, 95% A. Mass spectrometry was performed in full-scan mode over *m*/*z* 100–1000 using electrospray ionization (ESI). The source temperature was 500 °C, and the ion spray voltage was set to 5500 V in positive mode and − 4500 V in negative mode. Gas pressures were adjusted as follows: gas 1, 55 psi; gas 2, 60 psi; curtain gas, 25 psi. Collision-activated dissociation was set to high.

Raw data were processed using MExplorer Ultimate software. Peak extraction was performed using the centric MS method, followed by QC calibration and retention time alignment. Features with a relative standard deviation (RSD) > 30% in QC samples were excluded. Metabolite identification was conducted against the DICP, HMDB, GNPS, MoNA, MsDial, and Lipidblast databases. Metabolites with MS/MS spectral matches against the database met the criteria for confidence level 2 and were retained for further analysis. Differential metabolites were screened based on variable importance in projection (VIP) > 1.0, *P* < 0.05, and fold change (FC) > 1.5.

### 2.9 Data processing and statistical analysis

All experiments were performed in triplicate, and data were presented as mean ± standard error (SE). One-way analysis of variance (ANOVA) followed by Duncan's multiple range test (*P* < 0.05) was conducted using SPSS 26.0 (IBM, Armonk, NY, USA). Multivariate analyses, including principal component analysis (PCA), partial least squares discriminant analysis (PLS-DA), and volcano plot analysis, were performed using MetaboAnalyst 6.0 (https://www.metaboanalyst.ca) and Wei Sheng Xin (https://www.bioinformatics.com.cn) to assess sample variation and identify differential compounds.

## 3 Results and discussion

### 3.1 pH variation

During pickling, the pH values of both egg white and egg yolk exhibited an overall increasing trend (Fig. 1A). The pH of egg white rose rapidly during the early stage, increasing from 9.78 ± 0.11 to 11.43 ± 0.03 within 0–6 days, followed by a slight decline thereafter. In comparison, the pH of egg yolk increased steadily throughout the pickling process, rising sharply from 6.40 ± 0.11 to 10.28 ± 0.11 over 0–18 days before gradually leveling off in the later stage. The rapid increase in pH at the initial stage was primarily attributed to the fast penetration of sodium hydroxide into the egg driven by osmotic pressure (Xue et al., 2023). As egg white was directly exposed to the alkaline environment, its pH increased more rapidly. Conversely, the rise in egg yolk pH was initially delayed due to the barrier effect of the surrounding egg white, but gradually accelerated as alkali diffused inward over prolonged pickling. It is worth noting that the slight decrease in pH of egg white observed at the later stage may be related to the pore-blocking effect of copper ions, which hinders further alkali penetration (Luo et al., 2024; Xue et al., 2022). Meanwhile, the slower increase in egg yolk pH during the later stage may be associated with the gradual formation of a dense protein gel network, which limits further alkali diffusion to a certain extent (Yang et al., 2019).

**Fig. 1:**
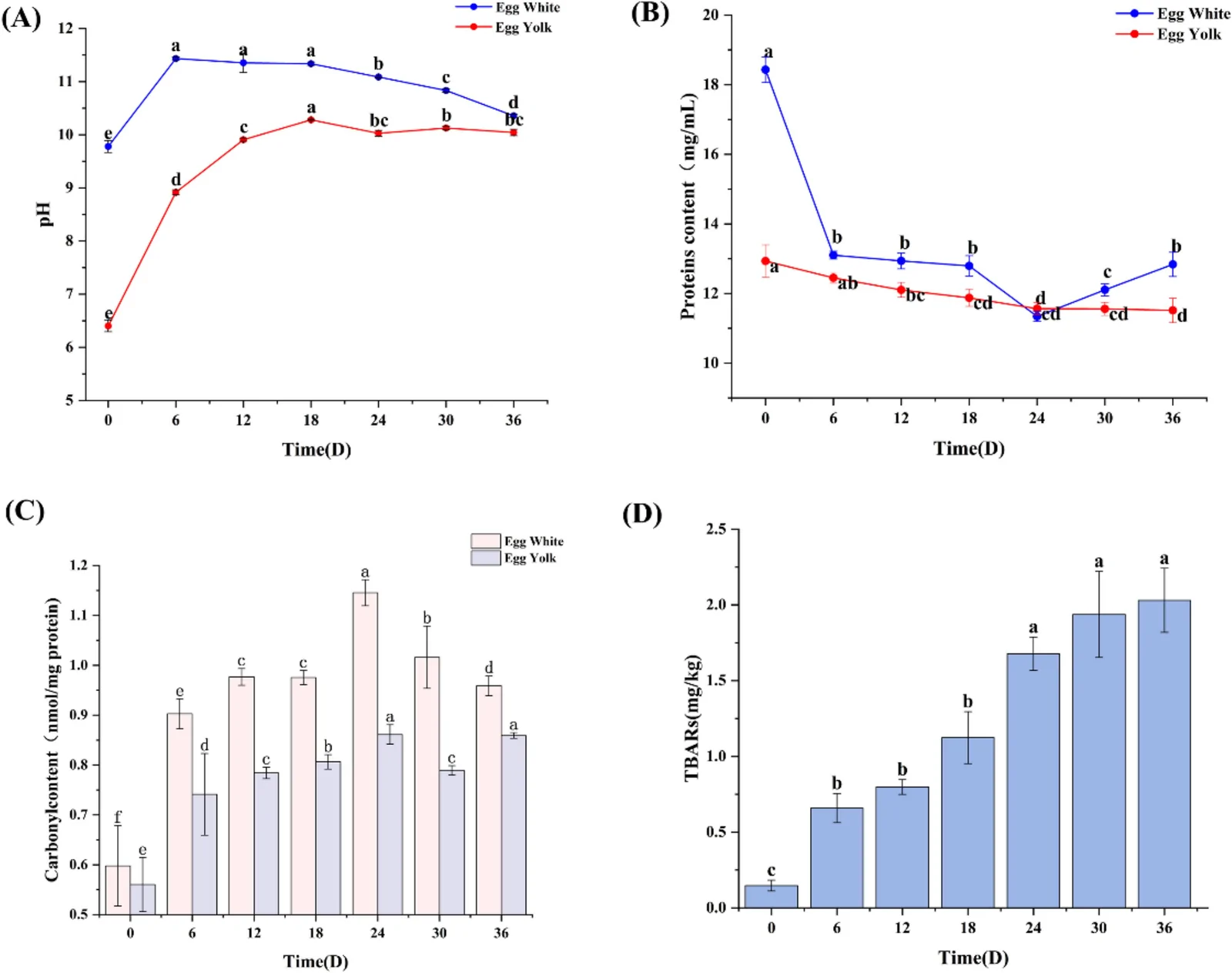
Changes in pH (A), protein content (B), and carbonyl content (C) of preserved egg white and yolk, and TBARS value (D) of preserved egg yolk during pickling.

### 3.2 Protein degradation and oxidation

Egg white protein content decreased significantly during the early stage, from 18.58 ± 0.37 mg/mL to 12.94 ± 0.11 mg/mL, and then declined more gradually before reaching a stable level (Fig. 1B). Egg yolk protein content also decreased continuously throughout the pickling period, from 12.93 ± 0.47 mg/mL to 11.51 ± 0.35 mg/mL, indicating that yolk lipoproteins underwent degradation under strong alkaline conditions. Protein degradation occurred primarily during the early stage, which may be attributed to the rapid pH increase promoting protein unfolding, thereby accelerating hydrolysis (Geng et al., 2026).

The carbonyl content, reflecting protein oxidation, increased significantly in both egg white and egg yolk during pickling (Fig. 1C). Egg white consistently displayed higher carbonyl levels than yolk, reaching a maximum of 1.15 ± 0.03 nmol/mg protein at 24 days. Egg yolk carbonyl content rose gradually, eventually stabilizing around 0.86 ± 0.01 nmol/mg protein. The continuous oxidation of proteins throughout the entire pickling process indicates that it is influenced by the duration of alkali treatment (Xiang et al., 2024). The differences in degradation and oxidation rates between egg white and yolk can be attributed to their distinct matrix compositions and structural characteristics. Overall, the stage-dependent degradation and oxidation of proteins are likely key factors driving the formation of characteristic flavor compounds in PEs.

### 3.3 Lipid oxidation

Egg yolk, rich in unsaturated fatty acids, was identified as the primary site of lipid oxidation during the pickling process. The TBARS value of egg yolk increased steadily over time, reaching an overall 12.79-fold increase by the end of pickling (Fig. 1D). The most pronounced rise occurred during the first 24 days, from 0.15 ± 0.04 mg/kg to 1.68 ± 0.11 mg/kg, whereas the increase between 24 and 36 days was only 21.00%, indicating that lipid oxidation mainly occurred during the early stage. This early-stage acceleration can be attributed to the initial strong alkaline conditions, which disrupts the egg yolk membrane structure and exposes large amounts of unsaturated fatty acids to oxidation. As the pickling process progresses, the alkalinity of the system gradually stabilizes, slowing down the rate of lipid oxidation and leading to a plateau (Moayedi et al., 2010; Xiang et al., 2024). Lipid oxidation was a key factor in the formation of volatile compounds in egg yolk; therefore, it is necessary to further investigate its contribution to the development of volatile compounds during the pickling process.

### 3.4 Flavor evolution of egg white during pickling

#### 3.4.1 Changes in volatile compounds of egg white during pickling

The changes in the flavor profile of egg white during pickling were investigated using an *E*-nose in combination with GC-IMS. The radar plot (Fig. 2A) illustrated the temporal changes in sensor responses, with sensors W2S, W5S, and W3S exhibiting relatively strong signals, suggesting that alcohols, aldehydes, ketones, alkanes, and nitrogen-containing compounds played important roles in flavor formation. Sensor responses varied across pickling stages, reflecting the gradual generation and transformation of volatile compounds. The PCA revealed distinct stage-dependent separation among samples **(**Fig. 2B), with PC1 and PC2 explaining 53.2% and 20.7% of the total variance, respectively (cumulative 73.9%). Early-stage samples (0–12 days) displayed the most pronounced differences, whereas later-stage samples (30 and 36 days) exhibited similar flavor profiles, indicating stabilization of the egg white aroma over time.

**Fig. 2:**
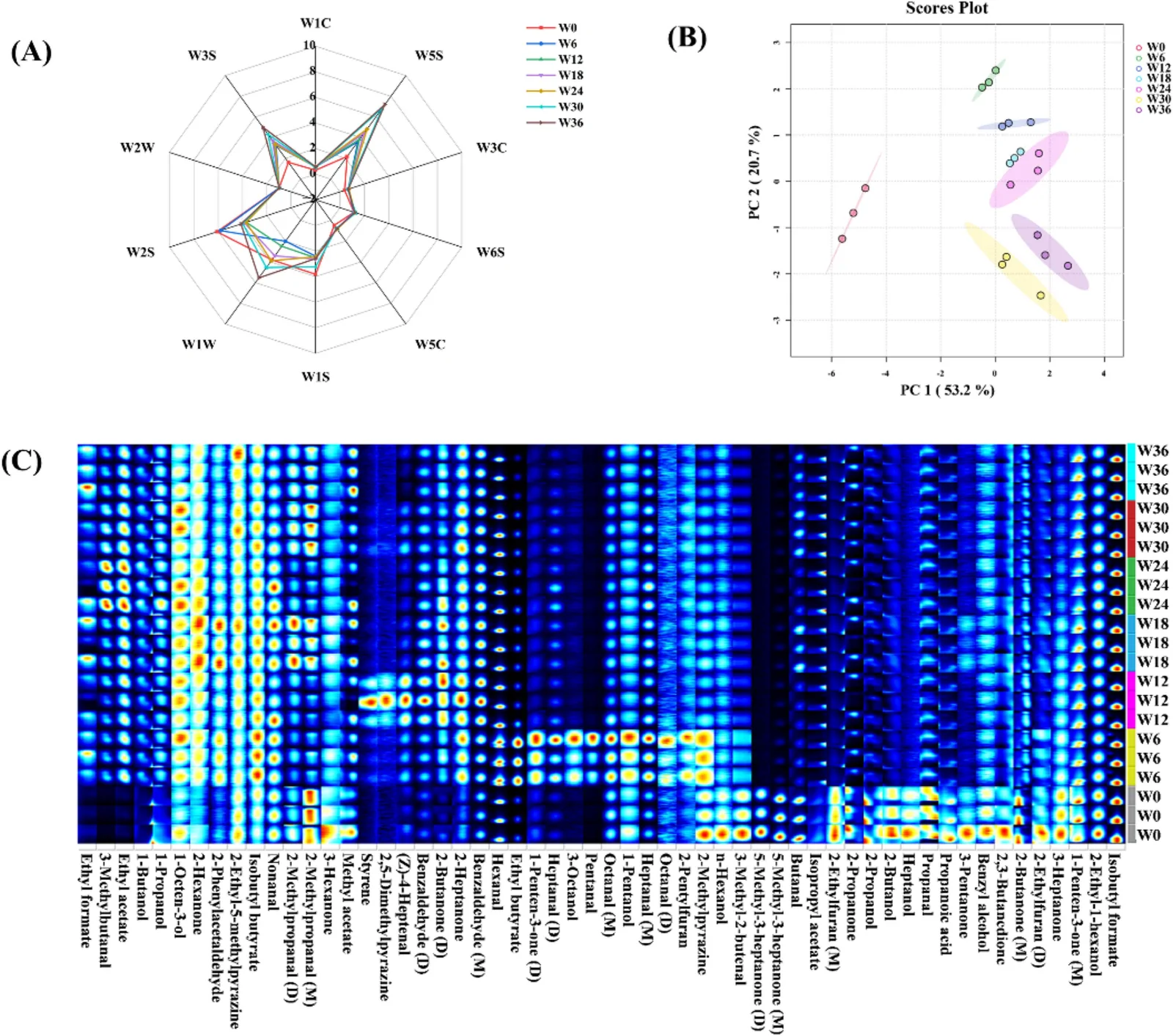
Changes in the flavor profile of egg white during pickling. (A) Radar plot of *E*-nose; (B) PCA score plot of E-nose; (C) Fingerprint plots of volatile compounds based on GC-IMS.

GC-IMS analysis identified 56 volatile compounds in egg white, including 17 aldehydes, 13 ketones, 11 alcohols, 7 esters, 1 acid, 3 furans, 3 nitrogen-containing compounds, and 1 other compound (Fig. 2C). Aldehydes, ketones, and alcohols were the predominant categories, which appeared to correspond to the sensitive responses of *E*-nose sensor W2S (**Table S1**). The flavor profile of egg white displayed clear stage-dependent characteristics. During the early stage (0–12 days), the profile was dominated by ketones (3-heptanone, 2-butanone (M), 5-methyl-3-heptanone (M/D), 1-penten-3-one (D)), alcohols (2-ethyl-1-hexanol), and aldehydes (3-methyl-2-butenal, propanal, octanal (M)). Aldehydes, which exhibit low odor thresholds, contribute significantly to overall egg white flavor (C. Li et al., 2022). Alcohols and ketones are mainly formed via amino acid degradation and lipid oxidation, and they are also essential for the characteristic egg white aroma (Shen et al., 2024b). In the middle stage (18–24 days), ester compounds such as isopropyl acetate and ethyl acetate increased significantly, imparting pleasant fruity and floral notes and enriching flavor complexity. During the late stage (30–36 days), cumulative aldehydes, ketones, and alcohols, including 3-methylbutanal, 2-hexanone, 1-propanol, 1-octen-3-ol as well as nitrogen-containing compounds such as 2-ethyl-5-methylpyrazine, dominated the flavor profile, forming the characteristic aroma of egg white. The results from the *E*-nose (Fig. 2A) and GC-IMS analyses (Fig. 2C) suggested a stage-dependent evolution of the egg white flavor throughout the pickling process. To further identify the odor-active compounds, GC–MS analysis was subsequently performed.

#### 3.4.2 Evolution of key aroma compounds of egg white

The changes of volatile compounds in egg white during pickling were monitored using GC–MS, and 61 compounds were detected over the 36 days, including 7 aldehydes, 10 ketones, 14 alcohols, 2 esters, 6 acids, 10 aromatic compounds, 1 furan, 8 nitrogen-containing compounds, and 3 other compounds (Fig. 3**A** and **B**). The relative proportions of volatile compounds reflect their contributions to the overall flavor profile (Fig. 3C). In egg white, ketones and furans predominated at the beginning of pickling, whereas the relative contents of alcohols and nitrogen-containing compounds steadily increased throughout the entire pickling process, becoming the major flavor contributors in the later stage. Because odor thresholds vary considerably among compounds, concentration alone is insufficient to accurately represent aroma intensity. Thus, the odor activity value (OAV), calculated as the ratio of compound concentration to its corresponding odor threshold, was employed to evaluate sensory contribution. Typically, compounds with OAV ≥ 1 are considered to play a key role in aroma formation. **Tables S3** and **S4** summarize the semi-quantitative concentrations and OAV of volatile compounds identified during the monitoring of volatile flavor formation. Overall, 16 volatile compounds with OAV ≥ 1 were identified throughout the pickling process. Among these, the compounds that exhibited OAV ≥ 1 and VIP > 1 on the 36th day of pickling were defined as the key aroma compounds in preserved egg white, namely nonanal, 1-octen-3-ol, and phenethyl alcohol. Notably, 1-octen-3-ol exhibited an OAV as high as 1302, making it the highest contributor to the aroma of egg white. The VIP scores obtained from PLS-DA were used to evaluate the discriminant role of individual metabolites in the classification of different pickling stages (Fig. 3**D** and **E**). The PLS-DA model showed good stability and reliability, with R^2^Y and Q^2^ values of 0.993 and 0.953, respectively. Moreover, no overfitting was observed following 200 permutation tests (**Fig. S1A**). Based on VIP analysis (VIP > 1, *P* < 0.05), a total of 19 key volatile compounds were identified as being associated with distinct pickling stages. These included four alcohols (1-octen-3-ol, 2-ethylhexanol, phenethyl alcohol, hexadecanol), two aldehydes (nonanal, pentadecanal), three ketones (6-methyl-5-hepten-2-one, 2-undecanone, cis-Geranylacetone), three aromatic compounds (benzaldehyde, indole, naphthalene), five nitrogen-containing compounds (2,6-dimethylpyrazine, 2-methylpyrazine, 2,5-dimethylpyrazine, 2-ethyl-5-methylpyrazine, pyrazine), as well as 2-ethylhexyl acetate and acetal.

**Fig. 3:**
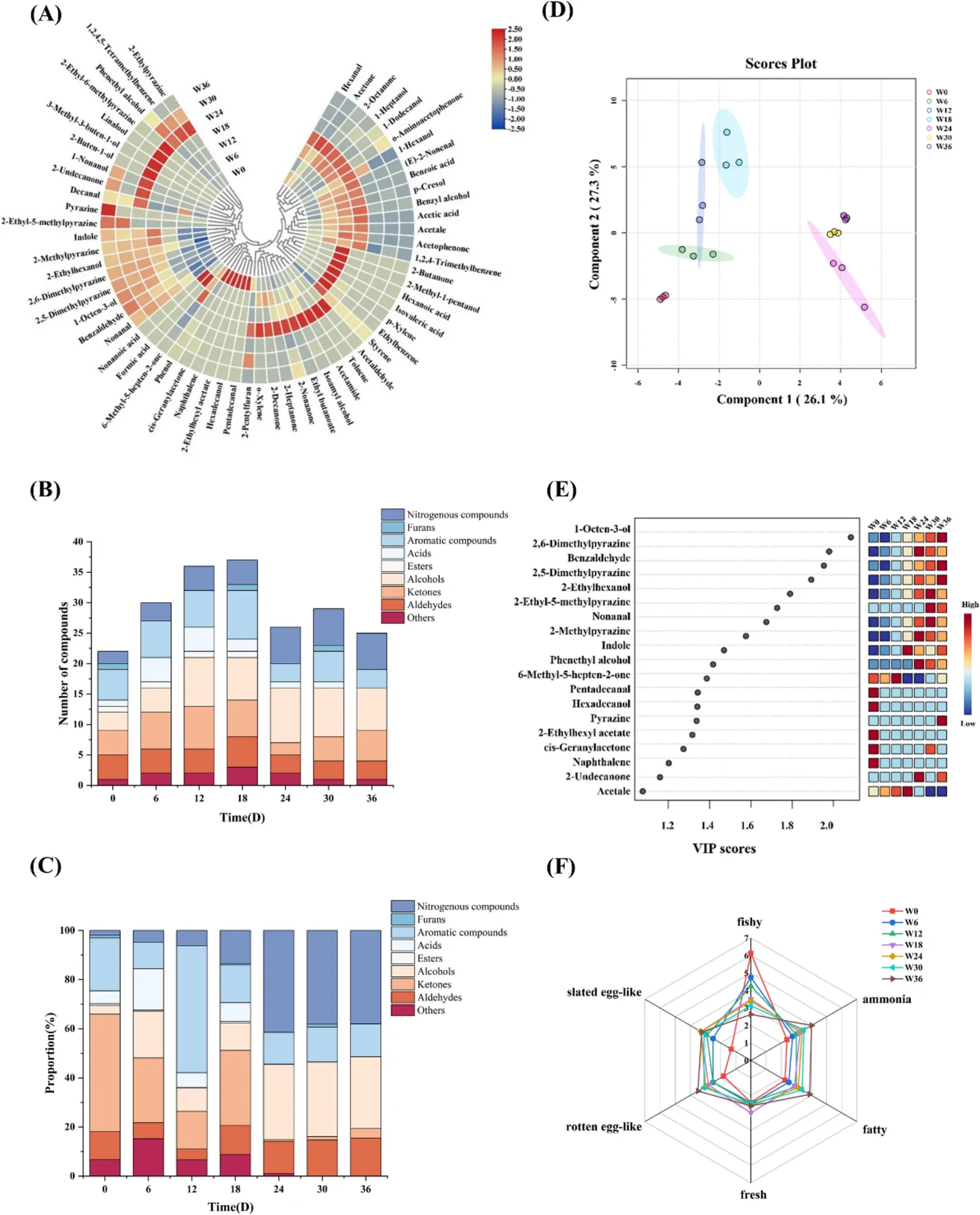
Volatile compounds in preserved egg white. (A) Heatmap of volatile compounds; (B) Changes in the number of volatile compound species over time; (C) Relative proportions of volatile compounds at different time points; (D) PLS-DA of volatile compounds at different stages; (E) Volatile compounds selected based on VIP scores (VIP > 1.0); (F) Radar plot of sensory evaluation scores. Data were obtained from GC–MS analysis and QDA.

1-Octen-3-ol is a common flavor compound generated from the transformation of linoleic acid, linolenic acid, or arachidonic acid (Zhu et al., 2024). This compound is abundant throughout the entire pickling process. Owing to its low sensory threshold and typical mushroom-like aroma, it contributes significantly to the flavor of egg white. Phenethyl alcohol, a typical product of phenylalanine degradation, appears on the 24th day of pickling and exhibits a rose-like sweet aroma, making a notable contribution to the flavor formation of egg white (Wang et al., 2026). Aldehydes with fewer than ten carbon atoms generally impart “green, fresh, and grassy” notes. As the carbon chain length increases, they may evoke a pronounced “oily” sensation (Zhao et al., 2025). Nonanal, commonly considered an oxidation and/or degradation product of ω-9 fatty acids, exhibits waxy and citrus-like notes and contributes significantly to the flavor development of egg white (Yu et al., 2023). Pentadecanal, primarily sourced from the oxidative degradation of fatty acids, imparts a fatty note to egg white and may originate from the influence of yolk flavor (Nie et al., 2025). In addition, benzaldehyde is generated during the pickling process, exhibiting bitter almond, cherry, and nutty notes. Its formation may result from the Strecker degradation of phenylalanine and the Maillard reaction (Z. Li et al., 2022). Esters are generally formed by the esterification of fats and proteins with acids (Shen et al., 2024a). The presence of 2-ethylhexyl acetate in the early stage of pickling indicates that esterification is relatively active during this period. Pyrazine compounds are primarily generated through lipid oxidation or the Maillard reaction. Most pyrazines are characterized by meaty and fried potato-like notes, and they significantly enhance the aroma and flavor of eggs (Li et al., 2023b).

As shown in Fig. 3F, quantitative descriptive analysis (QDA) revealed a marked shift in the flavor profile of preserved egg white during pickling. Fishy odor decreased progressively, whereas ammonia odor increased and became the dominant attribute at the end of pickling. Fatty and rotten egg-like odors also intensified gradually, while salted egg-like odor rose during the early stage and then plateaued. In contrast, fresh odor remained relatively stable throughout the process. These results indicated a transition of the egg white flavor from fresh to mature pickled. The fishy odor gradually diminished during pickling, likely due to precursor degradation and masking by newly formed volatiles. The ammonia odor was probably attributable to protein degradation and amino acid metabolism (Tang et al., 2025). This may also be related to the increased levels of nitrogen-containing compounds detected by GC–MS (Fig. 3A). It is noteworthy that the enhanced response of the *E*-nose W5S sensor may be correlated with the elevated ammonia odor intensity (Fig. 2A).

### 3.5 Flavor evolution of egg yolk during pickling

#### 3.5.1 Changes in volatile compounds of egg yolk during pickling

The combined analysis using an E-nose and GC-IMS revealed that the flavor profile of egg yolk underwent more complex and pronounced changes throughout the pickling process. Radar plots of E-nose responses (Fig. 4A) showed that sensors W5S and W1W exhibited the highest signals, indicating that nitrogen- and sulfur-containing compounds are the major volatile constituents in egg yolk. PCA based on sensor responses (Fig. 4B) was used to evaluate differences in yolk volatile composition across pickling stages. The degree of separation between data points reflects the extent of flavor variation, with marked separation indicating more significant differences. Samples from different pickling stages were clearly distinguished, demonstrating the continuous generation and conversion of volatile compounds throughout the pickling period. Compared with egg white, egg yolk exhibited stronger sensor responses, likely due to its higher lipid content, which promotes lipid oxidation and accelerates the formation of volatile compounds, ultimately resulting in substantially higher levels of volatile compounds in yolk than in egg white (Li et al., 2025).

**Fig. 4:**
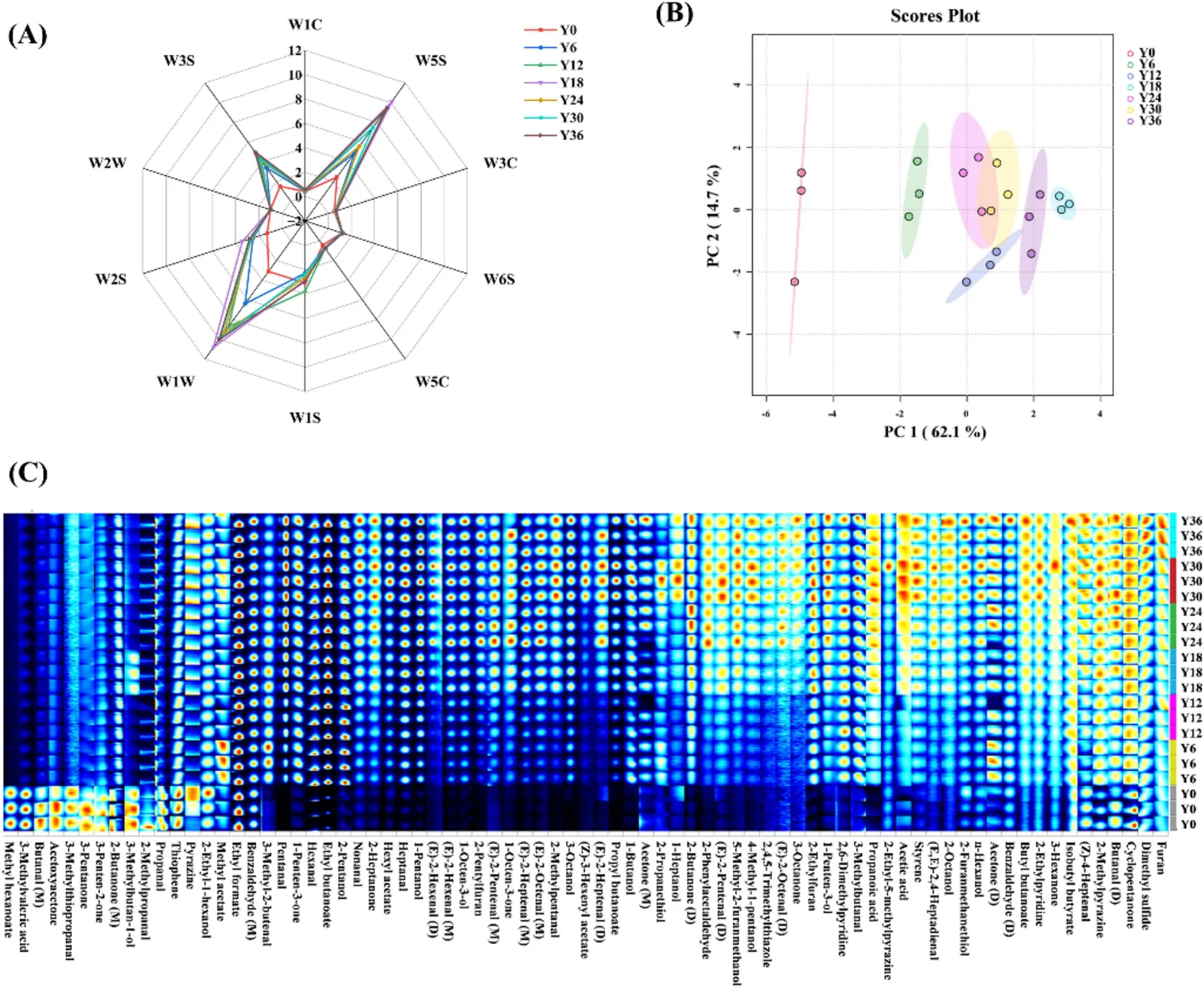
Changes in the flavor profile of egg yolk during pickling. (A) Radar plot of E-nose; (B) PCA score plot of E-nose; (C) Fingerprint plots of volatile compounds based on GC-IMS.

GC-IMS analysis identified 77 volatile compounds in egg yolk, including 25 aldehydes, 13 ketones, 15 alcohols, 9 esters, 3 acids, 3 furans, 5 nitrogen-containing compounds, and 4 other compounds (Fig. 4C). During pickling, alcohols including 1-hexanol, 2-octanol, 1-octen-3-ol, and 3-octanol increased progressively, imparting grassy, mushroom, and earthy notes. Aldehydes, mainly products of lipid oxidation, were more diverse and abundant than in egg white, including 2-methylpentanal, nonanal, (*E*)-2-heptenal (M/D), (*E*)-2-hexenal (M/D), (*E*)-2-pentenal (M/D), (*E*)-2-octenal (M/D), pentanal, heptanal, and benzaldehyde (D). Notably, heptanal and nonanal were identified as key aldehydes, with their concentrations gradually increasing over time. They contribute fatty, citrusy, and green notes to the egg yolk (Wang et al., 2025). Ketones such as 2-heptanone, 3-octanone, 2-butanone (D), 1-octen-3-one, and cyclopentanone accumulated steadily, forming characteristic yolk aroma. The detection of key esters, including butyl butanoate, propyl butanoate, hexyl acetate, and (Z)-3-hexenyl acetate, indicated active esterification during pickling. Short-chain esters typically impart a fruity aroma, whereas long-chain esters contribute fatty notes to the egg yolk (Zhou et al., 2025). Sulfur- and nitrogen-containing compounds, such as 2,4,5-trimethylthiazole, 2-pentylfuran, dimethyl sulfide, and pyrazines, are likely generated through the sulfur amino acid degradation and Maillard reactions, imparting characteristic sulfurous, roasted, and meaty aromas (Li et al., 2023b). The majority of volatile compounds in egg yolk continued to increase throughout the pickling process, indicating that the complex chemical reactions under strong alkaline conditions promote flavor development, which is consistent with previous studies (Ren et al., 2021).

Overall, the flavor profiles of egg yolk are more complex and intense than that of egg white, due to its higher fat content. While egg white flavor is dominated by a limited number of key volatile compounds, yolk flavor arises from the dynamic contributions of alcohols, aldehydes, ketones, esters, and sulfur- and nitrogen-containing compounds, ultimately forming a richer and more layered aroma profile.

#### 3.5.2 Evolution of key aroma compounds of egg yolk

The dynamic changes of volatile compounds in egg yolk during the pickling process were analyzed by GC–MS. A total of 86 volatile compounds were identified over the 36-day pickling period, including 23 aldehydes, 19 ketones, 14 alcohols, 3 esters, 5 acids, 9 aromatic compounds, 2 furans, 9 nitrogen-containing compounds, and 2 other compounds (Fig. 5A). Fig. 5B illustrated the dynamic changes in the number of each compound category. Among these, aldehydes, ketones, and alcohols were the dominant categories throughout the entire pickling process. The relative content proportions of volatile compounds (Fig. 5C) further emphasized their contributions to the overall flavor profile. Ketones were the core compounds driving flavor formation during the early stage. As pickling time progressed, the relative proportions of aldehydes and alcohols continuously increased, becoming the major flavor contributors in the later stage of pickling. **Table S5** summarizes the volatile compounds detected during flavor development, while **Table S6** presents their odor thresholds and OAVs. Throughout the pickling process, 39 compounds exhibited OAVs ≥1. Among these, 23 compounds with OAV ≥ 1 and VIP > 1 on the 36th day of pickling were defined as the key aroma compounds in preserved egg yolk. Aldehydes were the most abundant, comprising 10 compounds, followed by 5 ketones (2-heptanone, 2-octanone, 2-nonanone, 6-methyl-2-heptanone, 1-octen-3-one) and 5 alcohols (1-octen-3-ol, 1-heptanol, 1-octanol, (*E*)-2-octen-1-ol, phenethyl alcohol). Aromatic compounds, furans, and nitrogen-containing compounds each comprised a single compound. The PLS-DA results revealed a gradual change in flavor components throughout the pickling process (Fig. 5D). The PLS-DA model showed R^2^Y = 0.997 and Q^2^ = 0.989. The negative Q^2^-intercept from the permutation test (200 times) confirmed the reliability of the model (**Fig. S1B**). Based on VIP analysis (VIP > 1, *P* < 0.05), 42 volatile compounds were screened, which were mainly aldehydes, ketones, alcohols, and nitrogen-containing compounds (Fig. 5E).

**Fig. 5:**
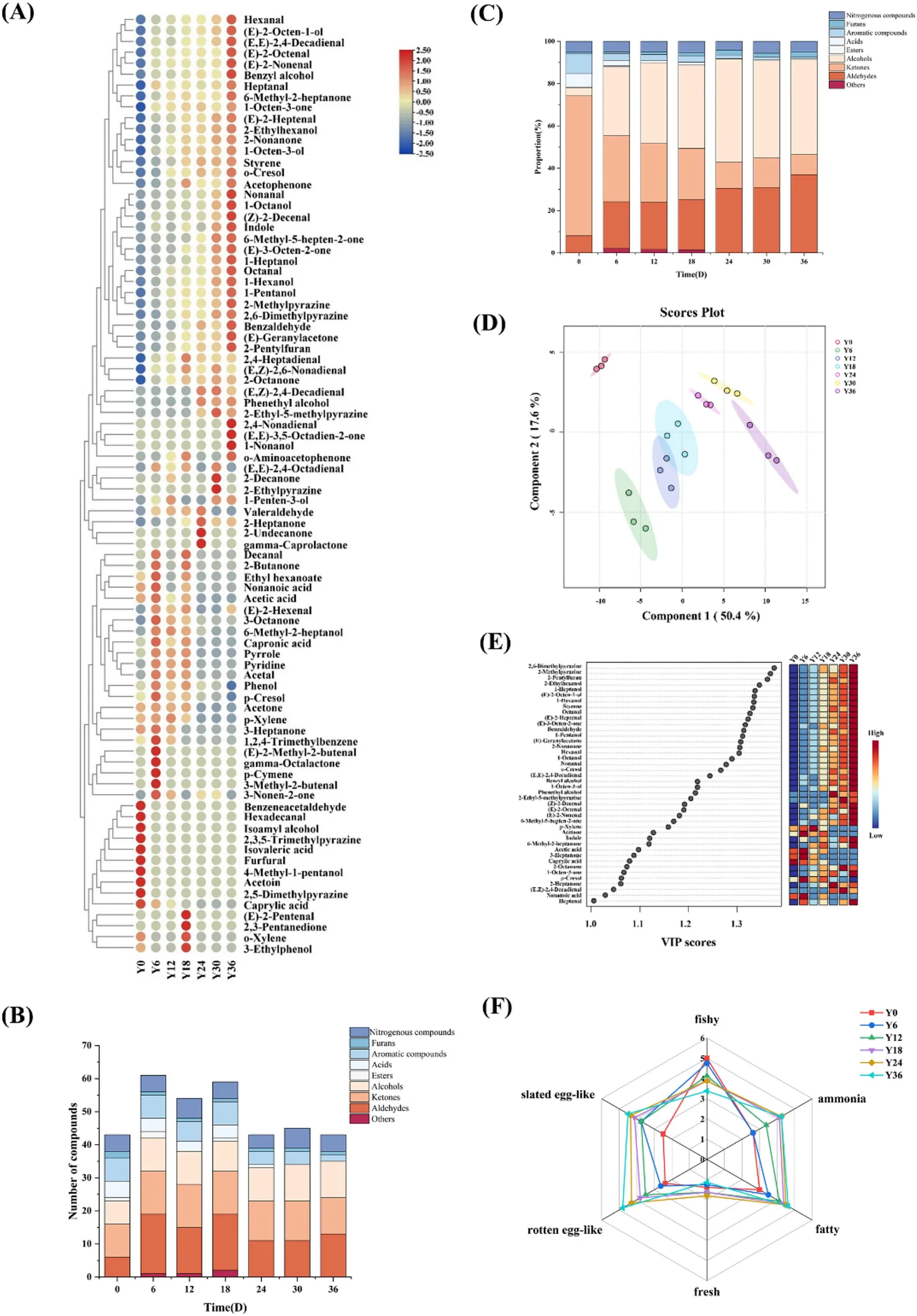
Volatile compounds in preserved egg yolk. (A) Heatmap of volatile compounds; (B) Changes in the number of volatile compound species over time; (C) Relative proportions of volatile compounds at different time points; (D) PLS-DA of volatile compounds at different stages; (E) Volatile compounds selected based on VIP scores (VIP > 1.0); (F) Radar plot of sensory evaluation scores. Data were obtained from GC–MS analysis and QDA.

Aldehydes are abundant flavor compounds in PEs, most of which are derived from lipid oxidation. It was observed that hexanal reached the highest concentration of 308.04 ± 26.07 ng/g, thus representing the predominant aldehyde. In egg yolk, hexanal, nonanal, octanal, heptanal, (*E*)-2-octenal, (*E*)-2-nonenal, and (*E,E*)-2,4-decadienal all exhibited OAV ≥ 1, contributing green, fatty, fruity and fresh aromas and playing a key role in the flavor of preserved egg yolk. Ketones serve as intermediates in the formation of heterocyclic compounds, thus contributing significantly to aroma formation (Gao et al., 2024). 1-Octen-3-one is an unsaturated ketone with an extremely low odor threshold (0.003 ng/g), exhibiting herbal and mushroom-like notes, and contributes significantly to the flavor profile. 2-nonanone significantly influences the aroma of meat and meat products, displaying fruity, sweet, waxy, grassy, coconut-like, and creamy notes, while 2-heptanone exhibits fruity and blue cheese-like flavors (Shen et al., 2023). Although the content of ketones in egg yolk was lower than that of aldehydes, they still play a positive role in flavor formation. Alcohols are typically generated from the oxidative decomposition of lipids. In egg yolk, in addition to 1-octen-3-ol and phenethyl alcohol, which have low odor thresholds and are contributing factors, 1-octanol, 1-heptanol, and (*E*)-2-octen-1-ol also impart waxy green, musty leafy, and green citrus notes, respectively, thereby enriching the aroma profile of egg yolk. Furthermore, 2-pentylfuran was also detected in egg yolk. This compound is generally generated from lipid oxidation in pickled products and, as a key aroma compound, enhances the richness of fruity notes (Shen et al., 2024b).

As shown in Fig. 5F, the intensities of ammonia, fatty, rotten egg-like, and salted egg-like notes in egg yolk increased progressively during pickling, and remained at higher levels than those in egg white. The fatty odor was likely associated with lipid oxidation products, such as aldehydes (Wang et al., 2025), whose contents increased during pickling **(**Fig. 5C**)**. The progressive intensification of the rotten egg-like odor may be attributed to the continuous degradation of proteins and the subsequent accumulation of sulfur-containing compounds (Zhang et al., 2024). This trend parallels the increase in sulfur-containing compounds detected by GC-IMS **(**Fig. 4C**)**. The fresh odor was probably masked by other more intense odors. Compared with egg white, egg yolk exhibited more pronounced fatty note, which can be attributed to its higher lipid content. The intensification of the rotten egg-like odor was likely associated with elevated levels of sulfur-containing compounds, which was consistent with the enhanced response of the *E*-nose W1W sensor **(**Fig. 4A**)**.

It should be noted that the volatile compound concentrations reported in this study are semi-quantitative estimates obtained using a single internal standard. The estimated concentrations may deviate from the actual contents due to differences in solid-phase microextraction efficiency, mass spectrometric detector response characteristics, and sample matrix effects. In addition, the odor thresholds used for OAV calculations were derived from the literature, which may vary considerably depending on the matrix and analytical conditions employed across different studies. Therefore, the key aroma compounds identified based on OAV analysis should not be regarded as definitive. Further validation through sensory analysis techniques, such as GC-O, aroma recombination experiments, and omission tests is required.

### 3.6 Comparison of volatile compounds in preserved eggs identified by GC–MS and GC-IMS

To exploit the complementary capabilities of GC–MS and GC-IMS, both analytical platforms were applied in this study for a comprehensive characterization of volatile compounds in preserved eggs. As shown in Fig. 6**A** and **C**, a total of 13 and 26 volatile compounds were commonly identified by GC–MS and GC-IMS in egg white and egg yolk, respectively. The compounds commonly detected in egg white included nonanal, hexanal, benzaldehyde, 2-butanone, acetone, 2-heptanone, 1-octen-3-ol, ethyl butyrate, 2-pentylfuran, 2-methylpyrazine, 2-ethyl-5-methylpyrazine, and styrene. In egg yolk, the commonly identified volatile compounds comprised 10 aldehydes, 5 ketones, 6 alcohols, as well as acetic acid, styrene, 2-pentylfuran, 2-ethyl-5-methylpyrazine and 2-methylpyrazine. Notably, in both egg white and egg yolk matrices, the number of volatile compounds identified by GC–MS was considerably higher than that detected by GC-IMS (Fig. 6**B** and **D**). The discrepancy can be attributed to the inherent differences between the two analytical platforms. GC–MS provides reliable structural identification through NIST spectral library matching, whereas its SPME pretreatment may incur losses of highly volatile compounds (Wang et al., 2020). In contrast, GC-IMS usually does not require elaborate sample preparation, enables sensitive detection of trace polar volatiles, and allows rapid acquisition of volatile fingerprints; however, its relatively limited compound database restricts the range of identifiable volatiles (Meng et al., 2026). Therefore, integrating GC–MS and GC-IMS provides complementary information for volatile compound analysis, thus providing a more comprehensive and reliable flavor characterization of complex food matrices such as preserved eggs.

**Fig. 6:**
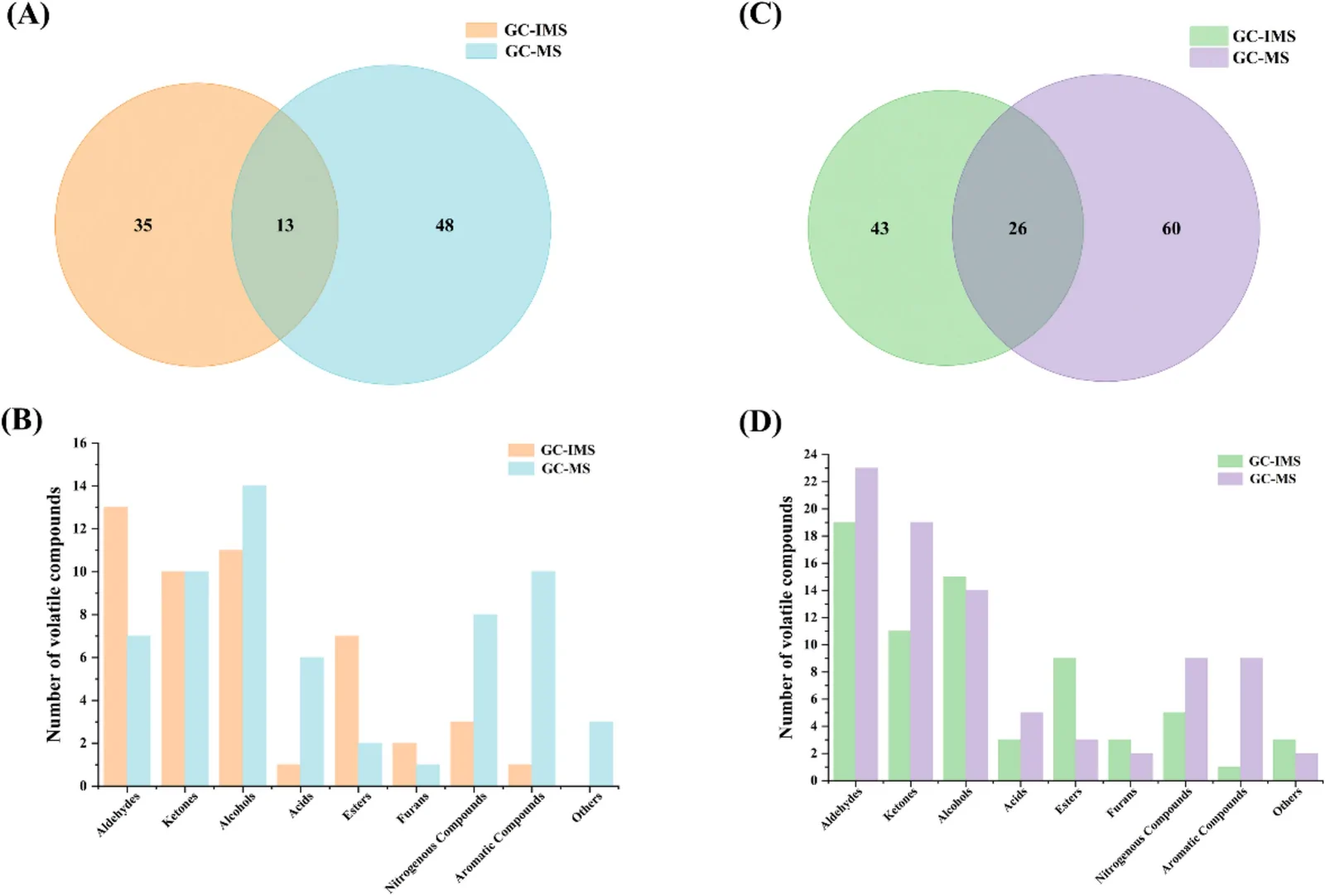
Venn diagram (A) and histogram (B) of volatile compounds identified by GC–MS and GC-IMS in preserved egg white. Venn diagram (C) and histogram (D) of volatile compounds identified by GC–MS and GC-IMS in preserved egg yolk.

### 3.7 Formation pathways of key aroma compounds

#### 3.7.1 Non-volatile components

In this study, a total of 144 and 539 non-volatile compounds were identified in egg white and egg yolk, respectively, under both positive and negative ion modes, and their relative abundances are presented in Fig. 7**A** and **B**. In egg white, the most abundant classes comprised 70 lipid and lipid-like molecules (48.61%), 14 organic acids and derivatives (9.72%), 11 amino acids and derivatives (7.64%), and 14 organic oxygen compounds (9.72%). In egg yolk, the predominant classes included 419 lipid and lipid-like molecules (77.74%), 30 amino acids and derivatives (5.57%), 13 benzenoids (2.41%), and 12 steroids (2.23%). PLS-DA score plots showed clear separation among samples collected at different pickling times, indicating that pickling duration strongly influenced the overall non-volatile compounds profiles (Fig. 7**C** and **D**). The PLS-DA model for egg white showed R^2^Y = 0.995 and Q^2^ = 0.61, and the model for egg yolk showed R^2^Y = 0.989 and Q^2^ = 0.654. Furthermore, the permutation test (200 times) confirmed the absence of overfitting (**Fig. S1C** and **S1D)**. To investigate differences in non-volatile compounds composition between fresh duck egg and preserved egg, comparisons were made between day 0 and day 36. Differential non-volatile compounds were screened using the criteria of VIP > 1.0, *P* < 0.05, and |FC| > 1.5. As shown in Fig. 7E, 51 differential non-volatile compounds were identified in egg white between day 0 and day 36, with 29 upregulated and 22 downregulated. In egg yolk, 149 differential non-volatile compounds were detected between day 0 and day 36, with 106 upregulated and 43 downregulated (Fig. 7F). These results demonstrated significant changes in non-volatile compounds during pickling. Differential non-volatile compounds in egg white were predominantly lipid and lipid-like molecules and organic acids and derivatives, whereas those in egg yolk were mainly lipid and lipid-like molecules and amino acids and derivatives.

**Fig. 7:**
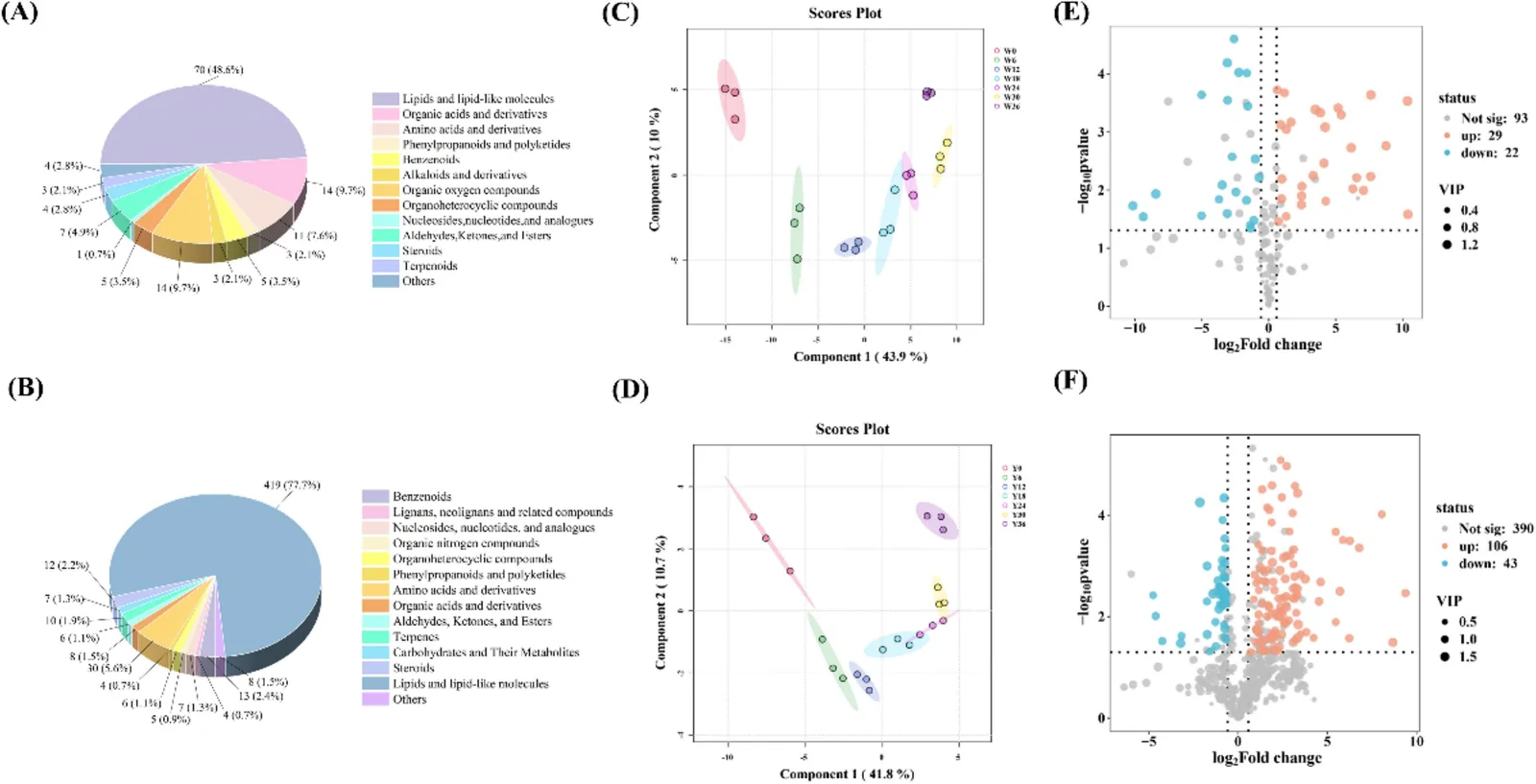
Non-volatile compounds of preserved egg white and yolk. Non-volatile compound classes in egg white (A) and egg yolk (B); PLS-DA score plots for egg white (C) and egg yolk (D); Volcano plots showing differential non-volatile compounds in egg white (E) and egg yolk (F). Data were obtained from LC-MS analysis.

It is well recognized that unsaturated fatty acids act as precursors for volatile compounds in high fat and high protein foods, and lipid oxidation has been shown to substantially contribute to the formation of characteristic flavor substances (Xiang et al., 2023). As key non-volatile compounds in the pickling process, lipids undergo oxidation during pickling, leading to the generation of unsaturated fatty acids such as linoleic acid and α-linolenic acid. These fatty acids can further undergo secondary reactions including oxidation and decomposition to form volatile compounds such as aldehydes, ketones, and alcohols (Chen et al., 2024). Additionally, amino acids are important contributors to taste and may be involved in the formation of flavor compounds. During pickling, they can undergo oxidative degradation and decarboxylation to form volatile compounds such as aldehydes and alcohols (Li et al., 2023a). Changes in carbonyl compounds and TBARS content further confirmed significant protein and lipid oxidation during the pickling of PEs.

#### 3.7.2 Correlation analysis between non-volatile and volatile compounds

To explore the molecular mechanisms of flavor formation in PEs, Spearman correlation analysis was conducted to elucidate the associations between non-volatile compounds and volatile compounds. In egg white, a total of 35 non-volatile compounds were found to be significantly correlated with 8 flavor compounds (|r| > 0.9, *P* < 0.001) (Fig. 8A). In egg yolk, 17 non-volatile compounds were identified as significantly correlated with 15 flavor compounds (|r| > 0.9, *P* < 0.001) (Fig. 8B). These results indicated strong associations between lipid metabolites, especially glycerophospholipids, and volatile compounds in both egg white and egg yolk. It has been reported that hydrolysis of phospholipids provides unsaturated fatty acids that readily undergo oxidation to form hydroperoxides (ROOH). Upon decomposition, ROOH generates reactive radicals capable of attacking fatty acids and inducing β-scission reactions, thereby producing a variety of volatile compounds, including aldehydes, ketones, and alcohols (Mariutti & Bragagnolo, 2017). For example, 2-heptanone and 1-octen-3-one can be produced via oxidative degradation of linoleic acid (Liu et al., 2023; Zhang et al., 2023), while 1-octen-3-ol and (*E*,*E*)-2,4-decadienal are probable secondary products formed in the autoxidation process of arachidonic acid and linoleic acid (Liu et al., 2024; Zhu et al., 2024). Among non-volatile compounds significantly correlated with aldehydes, glycerophospholipids accounted for the largest proportion, primarily including phosphatidylcholine (PC) and phosphatidylethanolamine (PE). Previous studies have suggested that the oxidation of phosphatidylcholine species, such as PC (18,0/18,2) and PC (18,0/18,1), might be associated with the formation of aldehydes such as octanal, nonanal, and hexanal (Zhao et al., 2025). Interestingly, 1-octen-3-ol in egg white was predominantly negatively correlated with PC, whereas (*E*)-2-octen-1-ol and 1-heptanol in egg yolk were mainly positively correlated with PC. In addition, ketones such as 2-heptanone, 2-nonanone, and 2-octanone showed strong positive correlations with glycerophospholipid metabolites. Under the non-enzymatic chemical reactions in a strongly alkaline environment, proteins were degraded into amino acids (Gao et al., 2022). These amino acids appear to undergo interconversion and show potential associations with flavor compound formation during pickling. Compounds such as 2-ethylhexanol and nonanal showed significant positive correlations with amino acid metabolites including Pro-Tyr-Arg-Glu and Asn-Phe-Ala-Arg. Fig. 8C illustrates the proposed pathways potentially involved in the formation of key aroma compounds. In addition to lipids metabolism, the Maillard reaction also showed close relationships to the flavor development of PEs. Amino acids and derivatives can also undergo Maillard reactions and Strecker degradation. In the Strecker degradation pathway, the amino acid reacts with an α-dicarbonyl compound to form a Schiff base, which is then decarboxylated and hydrolyzed to produce a Strecker aldehyde, accompanied by the reduction of the dicarbonyl compound to an aminoketone (El Hosry et al., 2025). The Maillard reaction between amino acids and reducing sugars can also generate pyrazines and other heterocyclic compounds, contributing to the roasted and fermented flavor characteristics of PEs (Nie et al., 2025). For example, L-threonine can be oxidized to 2,5-dimethylpyrazine via non-enzymatic reactions, and benzaldehyde is one of the oxidation products of phenylalanine and α-linolenic acid (Hu et al., 2024, Li et al., 2023a). Alkaline conditions favor the Maillard reaction and the generation of pyrazines. In the present study, the strong correlation of 2,6-dimethylpyrazine with D-4-hydroxyphenylglycine and d-Glucose in egg white indicates a potential link to the Maillard reaction. Nevertheless, this study mainly provided evidence for potential associations between metabolites and volatile compounds, while the direct causal relationships remain to be further validated, for instance via isotope-labeling approaches, while targeted pathway analysis is needed to verify the proposed mechanisms underlying flavor development.

**Fig. 8:**
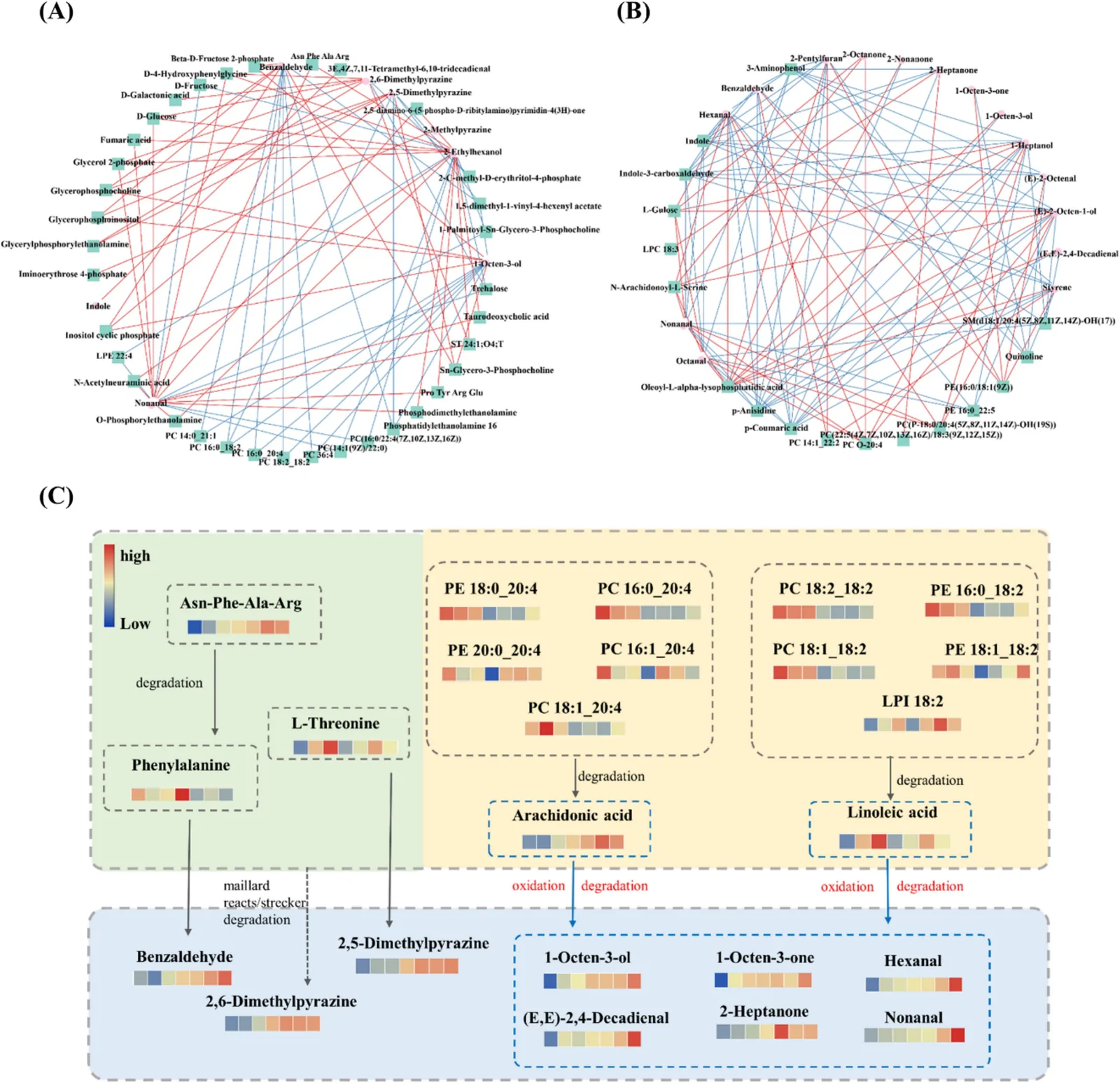
Correlation network analysis. Correlation networks between volatile compounds and differential non-volatile compounds in egg white (A) and egg yolk (B). Potential associations between non-volatile metabolites identified by LC-MS and key aroma compounds detected by GC–MS in PEs (C). The heatmap from left to right indicates the samples pickled for 0, 6, 12, 18, 24, 30 and 36 days.

## 4 Conclusion

In this study, a combined flavoromics and metabolomics strategy was adopted to explore the spatiotemporal evolution of volatile compounds in egg white and yolk during pickling. Results indicated that the early to mid-stage of pickling (0–24 days) was critical, with significant changes in pH, protein degradation, and lipid oxidation influencing final quality. Flavoromics analysis revealed clear differences in the types and levels of volatile compounds between egg white and yolk. The key aroma compounds were ide.

ntified in egg white, namely nonanal, 1-octen-3-ol, and phenethyl alcohol, while 23 key aroma compounds were identified in egg yolk, among which 1-octen-3-ol showed a relatively high contribution. Metabolomics analysis further indicated that lipid and amino acid related metabolites may contribute to flavor formation, and correlation analysis revealed potential associations between lipids and key aroma compounds. Integration of multi-omics data revealed potential formation pathways of key aroma compounds, but the contributions of protein and lipid degradation to flavor formation require further investigation. Overall, this study establishes a mechanistic and analytical foundation for the predictable modulation of preserved egg flavor during industrial production.

## CRediT authorship contribution statement

**Xiaoqian Chen:** Writing – original draft, Formal analysis, Data curation. **Ziyan Yue:** Writing – review & editing, Supervision, Methodology. **Yifei Li:** Methodology, Formal analysis. **Weixi Yang:** Methodology, Formal analysis. **Shugang Li:** Supervision. **Xiaobin Ma:** Validation. **Maurice O'Sullivan:** Validation. **Hong Zeng:** Writing – review & editing, Supervision, Methodology. **Yanbo Wang:** Supervision, Project administration, Methodology.

## Declaration of competing interest

The authors declare that they have no known competing financial interests or personal relationships that could have appeared to influence the work reported in this paper.

## Data Availability

Data will be made available on request.
