# Supplementary material for: Spatiotemporal evolution of aroma compounds in egg white and yolk of preserved eggs during pickling
Source: Food Chem X. 2026 Sep 19;39:104483. doi: 10.1016/j.fochx.2026.104483 (PMC13632092; doi:10.1016/j.fochx.2026.104483)
Supplement: Supplementary file 1 [file mmc1.docx]

**Table S1**

Information of 10 sensors of electronic nose of PEN3.5.

| **Sensor name** | **Representative species** | **Substances for sensing** |
| --- | --- | --- |
| W1C | Aromatic | Aromatic constituents, benzene |
| W5S | Broad range | Nitrogen oxides |
| W3C | Aromatic | Aroma, ammonia |
| W6S | Hydrogen | Hydrides |
| W5C | Arom-aliph | Alkane aromatic components |
| W1S | Broad-methane | Methyl, alkanes |
| W1W | Sulphur-organic | Sulphides |
| W2S | Broad-alcohol | Alcohols, aldehydes and ketones |
| W2W | Sulph-chlor | Organic sulphides |
| W3S | Methane-aliph | Alkanes |

**Table S2**

Attributes and compositions of the reference standards used in the sensory descriptive analyses of the preserved eggs.

| **Attribute** | **Reference standard** |
| --- | --- |
| Fishy | 2 g of grass carp gills and belly membrane were minced and placed in a covered 40 mL amber glass vial. |
| Ammonia | 1 mL of ammonium hydroxide solution (25%, w/w) was diluted in 500 mL of water and placed in a covered 40 mL amber glass vial. |
| Fatty | 2 g of finely chopped cooked pork belly (fatty portion) were placed in a covered 40 mL amber glass vial. |
| Fresh | 2 g of freshly cut cucumber pulp were minced and placed in a covered 40 mL amber glass vial. |
| Rotten egg-like | 2 g of freshly crushed rotten egg were placed in a covered 40 mL amber glass vial. |
| Salted egg-like | 2 g of cooked salted egg were mashed into a paste and placed in a covered 40 mL amber glass vial. |

**Table S3**

Semi-quantitative concentrations of volatile compounds in preserved egg white during pickling determined by GC-MS.

| No. | CAS | Compounds | RT(min) | RI^a^ | RI^b^ | ΔRI | SI | Concentrations(ng/g) | | | | | | |
| --- | --- | --- | --- | --- | --- | --- | --- | --- | --- | --- | --- | --- | --- | --- |
|  |  |  |  |  |  |  |  | 0d | 6d | 12d | 18d | 24d | 30d | 36d |
| 1 | 66-25-1 | Hexanal | 12.57 | 1084 | 1084 | 0 | 96 | 0.32±0.04^b^ | - | 0.36±0.03^b^ | 0.54±0.07^a^ | - | - | - |
| 2 | 124-19-6 | Nonanal | 23.46 | 1394 | 1397 | 3 | 99 | 2.86±1.25^b^ | 2.97±0.53^b^ | 5.43±0.92^ab^ | 6.17±0.5^a^ | 6.55±0.04^a^ | 6.94±0.17^a^ | 6.36±0.05^a^ |
| 3 | 2765-11-9 | Pentadecanal | 40.65 | 2043 | 2054 | 11 | 96 | 0.13±0.01^a^ | - | - | - | - | - | - |
| 4 | 100-52-7 | Benzaldehyde | 28.21 | 1542 | 1533 | 9 | 98 | 3.32±0.4^de^ | 2.15±0.09^e^ | 4.62±1.1^cd^ | 5.86±0.28^bc^ | 6.16±0.44^abc^ | 7.12±0.34^ab^ | 8.2±0.27^a^ |
| 5 | 112-31-2 | Decanal | 27.04 | 1505 | 1502 | 3 | 100 | - | 0.09±0.01^a^ | - | - | 0.11±0.07^a^ | 0.04±0.01^a^ | 0.05±0^a^ |
| 6 | 18829-56-6 | (*E*)-2-Nonenal | 28.33 | 1546 | 1547 | 1 | 96 | - | 0.16±0.02^a^ | 0.14±0.01^a^ | 0.22±0.04^a^ | - | - | - |
| 7 | 75-07-0 | Acetaldehyde | 4.73 | 678 | 690 | 12 | 98 | - | - | - | 16.43±7.41^a^ | - | - | - |
| 8 | 67-64-1 | Acetone | 5.86 | 805 | 800 | 5 | 98 | 25.24±0.45^b^ | 15.82±2.48^bc^ | 27.75±1.71^b^ | 62.51±11.37^a^ | - | - | 1.52±0.07^c^ |
| 9 | 110-93-0 | 6-Methyl-5-hepten-2-one | 21.68 | 1345 | 1347 | 2 | 86 | 0.65±0.07^b^ | 0.64±0.01^b^ | 1.02±0.15^a^ | - | - | 0.14±0.01^c^ | 0.22±0.02^c^ |
| 10 | 3796-70-1 | cis-Geranylacetone* | 37.57 | 1834 | 1813 | 21 | 98 | 0.83±0.08^a^ | - | - | - | - | 0.07±0.02^b^ | - |
| 11 | 98-86-2 | Acetophenone | 32.13 | 1671 | 1661 | 10 | 94 | 1.15±0.15^c^ | 1.85±0.14^b^ | 3.1±0.27^a^ | 2.7±0.1^a^ | 0.54±0.26^cd^ | 0.31±0.01^d^ | 0.46±0.08^cd^ |
| 12 | 110-43-0 | 2-Heptanone | 16.02 | 1183 | 1184 | 1 | 98 | - | 2.24±0.14^b^ | 1.05±0.06^bc^ | 4.82±0.95^a^ | - | - | - |
| 13 | 821-55-6 | 2-Nonanone | 23.35 | 1392 | 1393 | 1 | 95 | - | 0.71±0.03^c^ | 1.3±0.18^b^ | 3.51±0.21^a^ | - | 0.83±0.02^bc^ | 1.35±0.23^b^ |
| 14 | 693-54-9 | 2-Decanone | 26.79 | 1497 | 1497 | 0 | 93 | - | 0.45±0.04^b^ | - | 0.74±0.06^a^ | - | - | - |
| 15 | 78-93-3 | 2-Butanone | 7.33 | 898 | 899 | 1 | 96 | - | - | 1.54±0.04^b^ | - | - | - | - |
| 16 | 111-13-7 | 2-Octanone | 19.66 | 1286 | 1285 | 1 | 96 | - | - | 0.99±0.06^b^ | 1.43±0.15^a^ | - | - | - |
| 17 | 112-12-9 | 2-Undecanone | 30.19 | 1607 | 1602 | 5 | 86 | - | - | - | - | 0.2±0.02^a^ | - | 0.12±0.02^b^ |
| 18 | 111-27-3 | 1-Hexanol | 22.17 | 1360 | 1353 | 7 | 92 | 0.54±0.07^d^ | 0.79±0.03^c^ | 1.11±0.1^b^ | 1.71±0.06^a^ | 1.24±0.07^b^ | 0.16±0.02^e^ | 0.15±0^e^ |
| 19 | 3391-86-4 | 1-Octen-3-ol | 25.32 | 1453 | 1451 | 2 | 99 | 1.34±0.28^c^ | 1.31±0.04^c^ | 1.99±0.24^c^ | 3.79±0.19^b^ | 5.86±0.17^a^ | 6.01±0.29^a^ | 6.51±0.36^a^ |
| 20 | 36653-82-4 | Hexadecanol | 45.11 | 2385 | 2377 | 8 | 96 | 0.2±0.01^a^ | - | - | - | - | - | - |
| 21 | 104-76-7 | 2-Ethylhexanol | 26.62 | 1492 | 1490 | 2 | 99 | - | 12.99±0.22^c^ | 16.88±2.24^bc^ | 19.94±0.42^ab^ | 21.51±1.09^ab^ | 22.2±0.64^a^ | 20.67±1.42^ab^ |
| 22 | 100-51-6 | Benzyl alcohol | 38.26 | 1893 | 1884 | 9 | 90 | - | 0.57±0.05^b^ | 0.83±0.19^ab^ | 1.07±0.09^a^ | 0.14±0.01^c^ | 0.09±0^c^ | 0.15±0^c^ |
| 23 | 123-51-3 | Isoamyl alcohol | 17.41 | 1223 | 1211 | 12 | 96 | - | - | 0.16±0.05^b^ | 0.66±0.07^a^ | - | 0.05±0^c^ | - |
| 24 | 105-30-6 | 2-Methyl-1-pentanol | 20.35 | 1310 | 1317 | 7 | 99 | - | - | 1.23±0.41^a^ | - | - | - | - |
| 25 | 111-70-6 | 1-Heptanol | 25.53 | 1459 | 1459 | 0 | 80 | - | - | 0.2±0.02^a^ | 0.23±0.01^a^ | - | 0.04±0.01^b^ | - |
| 26 | 112-53-8 | 1-Dodecanol | 40.34 | 1971 | 1974 | 3 | 93 | - | - | 0.14±0.05^a^ | 0.18±0.03^a^ | - | 0.02±0^b^ | 0.04±0.01^b^ |
| 27 | 60-12-8 | Phenethyl alcohol | 39.20 | 1927 | 1920 | 7 | 96 | - | - | - | - | 0.62±0.11^a^ | 0.57±0.02^a^ | 0.23±0.1^b^ |
| 28 | 6117-91-5 | 2-Buten-1-ol | 17.09 | 1209 | 1193 | 16 | 81 | - | - | - | - | 0.14±0.03^a^ | - | - |
| 29 | 763-32-6 | 3-Methyl-3-buten-1-ol | 18.61 | 1253 | 1258 | 5 | 98 | - | - | - | - | 0.21±0.07^a^ | - | - |
| 30 | 78-70-6 | Linalool | 28.43 | 1551 | 1549 | 2 | 96 | - | - | - | - | 0.09±0.01^a^ | - | - |
| 31 | 143-08-8 | 1-Nonanol | 31.86 | 1663 | 1661 | 2 | 85 | - | - | - | - | 0.06±0.04^a^ | - | 0.03±0.01^a^ |
| 32 | 103-09-3 | 2-Ethylhexyl acetate* | 23.38 | 1389 | 1420 | 31 | 99 | 0.4±0.05^a^ | - | - | - | - | - | - |
| 33 | 105-54-4 | Ethyl butanoate | 11.04 | 1037 | 1036 | 1 | 93 | - | 0.33±0.06^c^ | 0.74±0.06^b^ | 1.75±0.09^a^ | - | - | - |
| 34 | 64-19-7 | Acetic acid | 25.85 | 1471 | 1461 | 10 | 95 | 3.07±1.1^ab^ | 9.56±0.93^ab^ | 10.01±5.89^ab^ | 14.94±3.83^a^ | 0.06±0.02^b^ | 0.11±0.02^b^ | - |
| 35 | 64-18-6 | Formic acid* | 28.11 | 1540 | 1501 | 39 | 96 | - | 1.31±1^a^ | - | - | - | - | - |
| 36 | 112-05-0 | Nonanoic acid | 45.37 | 2176 | 2169 | 7 | 94 | - | 0.08±0.04^a^ | - | - | - | - | - |
| 37 | 65-85-0 | Benzoic acid | 51.70 | 2462 | 2443 | 19 | 99 | - | 2.93±0.44^ab^ | 3.57±1.84^ab^ | 3.98±1.03^a^ | - | - | - |
| 38 | 503-74-2 | Isovaleric acid | 32.47 | 1683 | 1684 | 1 | 96 | - | - | 0.45±0.22^a^ | - | - | - | - |
| 39 | 142-62-1 | Hexanoic acid | 37.34 | 1857 | 1851 | 6 | 96 | - | - | 0.34±0.11^a^ | - | - | - | - |
| 40 | 106-42-3 | p-Xylene | 14.24 | 1131 | 1135 | 4 | 96 | 0.87±0.12^b^ | - | 38.54±6.52^a^ | 4.56±0.6^b^ | - | - | - |
| 41 | 95-47-6 | o-Xylene | 15.82 | 1178 | 1177 | 1 | 96 | - | 2.17±0.07^b^ | - | 5.05±0.9^a^ | - | - | - |
| 42 | 100-42-5 | Styrene | 18.72 | 1257 | 1261 | 4 | 95 | 3.62±0.72^a^ | 3.45±0.05^a^ | 22.05±11.75^a^ | 13.54±2.7^a^ | 11.27±0.4^a^ | 11.78±1.75^a^ | 11.3±0.95^a^ |
| 43 | 91-20-3 | Naphthalene | 35.91 | 1767 | 1753 | 14 | 96 | 1.7±0.56^a^ | - | - | - | - | - | - |
| 44 | 100-41-4 | Ethylbenzene | 13.90 | 1123 | 1124 | 1 | 96 | - | 1.12±0.02^b^ | 60.97±11.51^a^ | 4.54±0.77^b^ | - | - | - |
| 45 | 108-88-3 | Toluene | 11.24 | 1043 | 1043 | 0 | 96 | - | - | - | 7.37±0.66^a^ | - | - | - |
| 46 | 108-95-2 | Phenol | 41.69 | 2024 | 2015 | 9 | 89 | 5.65±5.1^a^ | 0.33±0.17^a^ | 0.68±0.17^a^ | 0.86±0.11^a^ | 1.32±0.15^a^ | 1.19±0.01^a^ | 1.14±0.03^a^ |
| 47 | 106-44-5 | p-Cresol | 43.57 | 2100 | 2092 | 8 | 95 | - | 0.36±0.03^b^ | 0.5±0.08^ab^ | 0.55±0.04^a^ | - | 0.05±0.01^c^ | - |
| 48 | 95-63-6 | 1,2,4-Trimethylbenzene | 19.50 | 1277 | 1284 | 7 | 98 | 0.7±0.08^c^ | 1.42±0.17^ab^ | 1.21±0.08^b^ | 1.69±0.15^a^ | - | 0.41±0.01^cd^ | 0.24±0.01^de^ |
| 49 | 95-93-2 | 1,2,4,5-Tetramethylbenzene | 25.19 | 1450 | 1430 | 20 | 85 | - | - | - | - | 0.11±0.06^a^ | 0.07±0^ab^ | - |
| 50 | 3777-69-3 | 2-Pentylfuran | 17.29 | 1220 | 1220 | 1 | 88 | 0.64±0.06^c^ | - | - | 1.4±0.11^a^ | - | 1.2±0.03^b^ | - |
| 51 | 123-32-0 | 2,5-Dimethylpyrazine | 21.41 | 1337 | 1324 | 13 | 95 | 0.57±0.07^d^ | 1.36±0.24^c^ | 1.4±0.21^c^ | 3.04±0.4^b^ | 3.72±0.12^ab^ | 3.53±0.03^ab^ | 3.8±0.02^a^ |
| 52 | 108-50-9 | 2,6-Dimethylpyrazine | 21.61 | 1345 | 1331 | 14 | 96 | - | 1.97±0.25^c^ | 9.11±1.42^b^ | 19.68±0.53^a^ | 23.85±0.2^a^ | 22.89±2.37^a^ | 22.45±2.75^a^ |
| 53 | 109-08-0 | 2-Methylpyrazine | 19.49 | 1282 | 1276 | 6 | 97 | - | - | 3.37±1.7^bc^ | 8.89±2.41^a^ | 6.88±0.04^ab^ | 6.85±0.07^ab^ | 6.24±1.59^ab^ |
| 54 | 13925-00-3 | 2-Ethylpyrazine | 21.66 | 1344 | 1336 | 8 | 92 | - | - | - | - | 0.6±0.26^a^ | 0.3±0^ab^ | - |
| 55 | 13360-64-0 | 2-Ethyl-5-methylpyrazine | 23.37 | 1332 | 1324 | 8 | 98 | - | - | - | - | - | 1.77±0.1^a^ | 1.72±0.04^a^ |
| 56 | 13925-03-6 | 2-Ethyl-6-methylpyrazine | 23.38 | 1337 | 1331 | 6 | 97 | - | - | - | - | 4.08±1.67^a^ | - | - |
| 57 | 290-37-9 | Pyrazine | 17.43 | 1222 | 1217 | 5 | 96 | - | - | - | - | - | - | 0.46±0.03^a^ |
| 58 | 120-72-9 | Indole | 52.05 | 2480 | 2463 | 17 | 95 | 0.55±0.06^c^ | 0.62±0.02^c^ | 1.06±0.31^bc^ | 1.85±0.13^a^ | 1.43±0.16^ab^ | 1.33±0.09^ab^ | 1.5±0.17^ab^ |
| 59 | 105-57-7 | Acetale | 7.08 | 881 | 892 | 11 | 95 | 3.89±0.19^c^ | 11.63±0.72^b^ | 15.68±2.18^a^ | 17.66±0.99^a^ | 0.99±0.2^c^ | - | - |
| 60 | 60-35-5 | Acetamide | 35.03 | 1773 | 1775 | 2 | 93 | - | 0.92±0.47^b^ | - | 4.05±1.22^a^ | - | - | - |
| 61 | 551-93-9 | o-Aminoacetophenone | 47.03 | 2249 | 2252 | 3 | 83 | - | - | 0.26±0.06^a^ | 0.31±0.02^a^ | 0.05±0.01^bc^ | 0.03±0^bc^ | 0.13±0.03^b^ |

^a^: Retention indices (RI) calculated based on n-alkanes (C7-C34). ^b^: Retention indices (RI) were referred to NIST database(<https://webbook.nist.gov/chemistry/>). RT, retention time (min). SI, similarity index. Compounds were identified by NIST library matching (similarity ≥ 80%) and retention index (RI) comparison with literature values. ΔRI ≤ 20: definitively identified; ΔRI 20-50: tentatively identified. *Compounds marked with an asterisk exhibited ΔRI of 20-50 and were regarded as tentatively identified. ‘-’, no data found.

**Table S4**

Odor activity values (OAV) of volatile compounds in preserved egg white during pickling.

| No. | CAS | Compounds | OT(ng/g)^a^ | OAV | | | | | | |
| --- | --- | --- | --- | --- | --- | --- | --- | --- | --- | --- |
|  |  |  |  | 0d | 6d | 12d | 18d | 24d | 30d | 36d |
| 1 | 66-25-1 | Hexanal | 0.32 | ＜1 | - | 1 | 1 | - | - | - |
| 2 | 124-19-6 | Nonanal | 1 | 2 | 2 | 5 | 6 | 6 | 6 | 6 |
| 3 | 2765-11-9 | Pentadecanal | 430 | ＜1 | - | - | - | - | - | - |
| 4 | 100-52-7 | Benzaldehyde | 24 | ＜1 | ＜1 | ＜1 | ＜1 | ＜1 | ＜1 | ＜1 |
| 5 | 112-31-2 | Decanal | 0.08 | - | 1 | - | - | 1 | ＜1 | ＜1 |
| 6 | 18829-56-6 | (*E*)-2-Nonenal | 0.08 | - | 1 | 1 | 2 | - | - | - |
| 7 | 75-07-0 | Acetaldehyde | 0.7 | - | - | - | 23 | - | - | - |
| 8 | 67-64-1 | Acetone | 40 | ＜1 | ＜1 | ＜1 | 1 | - | - | ＜1 |
| 9 | 110-93-0 | 6-Methyl-5-hepten-2-one | 50 | ＜1 | ＜1 | ＜1 | - | - | ＜1 | ＜1 |
| 10 | 3796-70-1 | cis-Geranylacetone | 60 | ＜1 | - | - | - | - | ＜1 | - |
| 11 | 98-86-2 | Acetophenone | 36 | ＜1 | ＜1 | ＜1 | ＜1 | ＜1 | ＜1 | ＜1 |
| 12 | 110-43-0 | 2-Heptanone | 1 | - | 2 | 1 | 4 | - | - | - |
| 13 | 821-55-6 | 2-Nonanone | 5 | - | ＜1 | ＜1 | ＜1 | - | ＜1 | ＜1 |
| 14 | 693-54-9 | 2-Decanone | 3 | - | ＜1 | - | ＜1 | - | - | - |
| 15 | 78-93-3 | 2-Butanone | 7000 | - | - | ＜1 | - | - | - | - |
| 16 | 111-13-7 | 2-Octanone | 5 | - | - | ＜1 | ＜1 | - | - | - |
| 17 | 112-12-9 | 2-Undecanone | 5.5 | - | - | - | - | ＜1 | - | ＜1 |
| 18 | 111-27-3 | 1-Hexanol | 5.6 | ＜1 | ＜1 | ＜1 | ＜1 | ＜1 | ＜1 | ＜1 |
| 19 | 3391-86-4 | 1-Octen-3-ol | 0.005 | 267 | 262 | 398 | 757 | 1172 | 12＜12 | 13＜12 |
| 20 | 36653-82-4 | Hexadecanol | 1100 | ＜1 | - | - | - | - | - | - |
| 21 | 104-76-7 | 2-Ethylhexanol | 198 | - | ＜1 | ＜1 | ＜1 | ＜1 | ＜1 | ＜1 |
| 22 | 100-51-6 | Benzyl alcohol | 100 | - | ＜1 | ＜1 | ＜1 | ＜1 | ＜1 | ＜1 |
| 23 | 123-51-3 | Isoamyl alcohol | 4 | - | - | ＜1 | ＜1 | - | ＜1 | - |
| 24 | 105-30-6 | 2-Methyl-1-pentanol | 830 | - | - | ＜1 | - | - | - | - |
| 25 | 111-70-6 | 1-Heptanol | 5.4 | - | - | ＜1 | ＜1 | - | ＜1 | - |
| 26 | 112-53-8 | 1-Dodecanol | 16 | - | - | ＜1 | ＜1 | - | ＜1 | ＜1 |
| 27 | 60-12-8 | Phenethyl alcohol | 0.015 | - | - | - | - | 41 | 37 | 15 |
| 28 | 6117-91-5 | 2-Buten-1-ol | - | - | - | - | - | - | - | - |
| 29 | 763-32-6 | 3-Methyl-3-buten-1-ol | 547.125 | - | - | - | - | ＜1 | - | - |
| 30 | 78-70-6 | Linalool | 0.01 | - | - | - | - | 8 | - | - |
| 31 | 143-08-8 | 1-Nonanol | 34 | - | - | - | - | ＜1 | - | ＜1 |
| 32 | 103-09-3 | 2-Ethylhexyl acetate | 43 | ＜1 | - | - | - | - | - | - |
| 33 | 105-54-4 | Ethyl butanoate | 0.001 | - | 333 | 735 | 1748 | - | - | - |
| 34 | 64-19-7 | Acetic acid | 10000 | ＜1 | ＜1 | ＜1 | ＜1 | 5 | ＜1 | - |
| 35 | 64-18-6 | Formic acid | 46000 | - | ＜1 | - | - | - | - | - |
| 36 | 112-05-0 | Nonanoic acid | 34 | - | ＜1 | - | - | - | - | - |
| 37 | 65-85-0 | Benzoic acid | 1000 | - | ＜1 | ＜1 | ＜1 | - | - | - |
| 38 | 503-74-2 | Isovaleric acid | 12 | - | - | ＜1 | - | - | - | - |
| 39 | 142-62-1 | Hexanoic acid | 35.6 | - | - | ＜1 | - | - | - | - |
| 40 | 106-42-3 | p-Xylene | 530 | ＜1 | - | ＜1 | ＜1 | - | - | - |
| 41 | 95-47-6 | o-Xylene | 450.23 | - | ＜1 | - | ＜1 | - | - | - |
| 42 | 100-42-5 | Styrene | 3.6 | 1 | ＜1 | 6 | 3 | 3 | 3 | 3 |
| 43 | 91-20-3 | Naphthalene | 1 | 1 | - | - | - | - | - | - |
| 44 | 100-41-4 | Ethylbenzene | 2.4 | - | ＜1 | 25 | 1 | - | - | - |
| 45 | 108-88-3 | Toluene | 24 | - | - | - | ＜1 | - | - | - |
| 46 | 108-95-2 | Phenol | 31 | ＜1 | ＜1 | ＜1 | ＜1 | ＜1 | ＜1 | ＜1 |
| 47 | 106-44-5 | p-Cresol | 1 | - | ＜1 | ＜1 | ＜1 | - | ＜1 | - |
| 48 | 95-63-6 | 1,2,4-Trimethylbenzene | 260 | ＜1 | ＜1 | ＜1 | ＜1 | - | ＜1 | ＜1 |
| 49 | 95-93-2 | 1,2,4,5-Tetramethylbenzene | - | - | - | - | - | - | - | - |
| 50 | 3777-69-3 | 2-Pentylfuran | 5.8 | ＜1 | - | - | ＜1 | - | ＜1 | - |
| 51 | 123-32-0 | 2,5-Dimethylpyrazine | 80 | ＜1 | ＜1 | ＜1 | ＜1 | ＜1 | ＜1 | ＜1 |
| 52 | 108-50-9 | 2,6-Dimethylpyrazine | 400 | - | ＜1 | ＜1 | ＜1 | ＜1 | ＜1 | ＜1 |
| 53 | 109-08-0 | 2-Methylpyrazine | 60 | - | - | ＜1 | ＜1 | ＜1 | ＜1 | ＜1 |
| 54 | 13925-00-3 | 2-Ethylpyrazine | 1000 | - | - | - | - | ＜1 | ＜1 | - |
| 55 | 13360-64-0 | 2-Ethyl-5-methylpyrazine | 16 | - | - | - | - | - | ＜1 | ＜1 |
| 56 | 13925-03-6 | 2-Ethyl-6-methylpyrazine | 40 | - | - | - | - | ＜1 | - | - |
| 57 | 290-37-9 | Pyrazine | 75000 | - | - | - | - | - | - | ＜1 |
| 58 | 120-72-9 | Indole | 11 | ＜1 | ＜1 | ＜1 | ＜1 | ＜1 | ＜1 | ＜1 |
| 59 | 105-57-7 | Acetale | 4.9 | ＜1 | 2 | 3 | 3 | ＜1 | - | - |
| 60 | 60-35-5 | Acetamide | 140000 | - | 6 | - | ＜1 | - | - | - |
| 61 | 551-93-9 | o-Aminoacetophenone | 0.2 | - | - | 1 | 1 | ＜1 | ＜1 | ＜1 |

^a^: Odor thresholds were referenced as a published book, named: Odor thresholds-compilations of odor threshold values in air, water and other media (2nd ed). ‘-’, no data found.

Note: Volatile quantification data for OAV calculation were determined using GC-MS.

**Table S5**
Semi-quantitative concentrations of volatile compounds in preserved egg yolk during pickling determined by GC-MS.

| No. | CAS | Compounds | RI^a^ | RI^b^ | RT | ΔRI | SI | Concentrations(ng/g) | | | | | | |
| --- | --- | --- | --- | --- | --- | --- | --- | --- | --- | --- | --- | --- | --- | --- |
|  |  |  |  |  |  |  |  | 0d | 6d | 12d | 18d | 24d | 30d | 36d |
| 1 | 66-25-1 | Hexanal | 1084 | 1084 | 12.59 | 0 | 95 | 5.31±0.77^d^ | 109.93±11.98^c^ | 130.56±6.8^c^ | 148.51±11.5^bc^ | 156.92±10.72^bc^ | 193.91±15.62^b^ | 308.04±26.07^a^ |
| 2 | 124-19-6 | Nonanal | 1401 | 1397 | 23.51 | 4 | 98 | 2.48±0.74^e^ | 18.32±1.9^de^ | 26.3±2.87^cd^ | 36.08±5.89^cd^ | 40.5±2.39^c^ | 74.26±4.57^b^ | 141.62±7.06^a^ |
| 3 | 112-31-2 | Decanal | 1507 | 1502 | 27.14 | 5 | 90 | 0.42±0.16^b^ | 3.03±0.35^a^ | - | 3.2±0.45^a^ | - | - | 0±0^b^ |
| 4 | 100-52-7 | Benzaldehyde | 1544 | 1533 | 28.21 | 11 | 98 | 12.9±1.06^e^ | 13.28±2.6^e^ | 15.31±1.05^e^ | 30.09±2.11^d^ | 48.59±0.67^b^ | 39.34±2.86^c^ | 68.16±2.63^a^ |
| 5 | 122-78-1 | Benzeneacetaldehyde | 1664 | 1654 | 28.26 | 10 | 90 | 4.39±0.32^a^ | - | - | - | - | - | - |
| 6 | 629-80-1 | Hexadecanal | 2147 | 2141 | 44.68 | 6 | 95 | 0.97±0.05^a^ | - | - | - | - | - | - |
| 7 | 110-62-3 | Valeraldehyde | 980 | 980 | 9.367 | 0 | 98 | - | 11.95±2^b^ | 14.03±0.92^ab^ | 14.01±2.98^ab^ | 18.81±0.61^a^ | - | - |
| 8 | 497-03-0 | (*E*)-2-Methyl-2-butenal | 1101 | 1092 | 13.12 | 9 | 93 | - | 6.03±0.22^a^ | - | - | - | - | - |
| 9 | 111-71-7 | Heptanal | 1189 | 1188 | 16.05 | 1 | 90 | - | 15.68±2.68^b^ | 12.64±0.49^b^ | 13.93±0.92^b^ | 11.79±1.09^b^ | 14.69±0.68^b^ | 21.29±0.84^a^ |
| 10 | 107-86-8 | 3-Methyl-2-butenal | 1212 | 1206 | 17.07 | 6 | 88 | - | 1.76±0.26^a^ | - | - | - | - | - |
| 11 | 6728-26-3 | (*E*)-2-Hexenal | 1228 | 1222 | 17.58 | 6 | 88 | - | 2±0.23^a^ | 1.43±0.06^a^ | 1.93±0.29^a^ | - | - | 1.46±0.08^a^ |
| 12 | 124-13-0 | Octanal | 1294 | 1292 | 19.82 | 2 | 97 | - | 7.67±1.07^c^ | 12.05±0.18^c^ | 12.21±1.63^c^ | 12.83±0.84^c^ | 20.5±1.83^b^ | 29.96±2.22^a^ |
| 13 | 18829-55-5 | (*E*)-2-Heptenal | 1335 | 1328 | 21.32 | 7 | 76 | - | 10.12±0.8^c^ | 11.44±1.58^c^ | 15.31±1.89^bc^ | 15.36±1.58^bc^ | 19.57±1.6^ab^ | 23.13±0.21^a^ |
| 14 | 2548-87-0 | (*E*)-2-Octenal | 1441 | 1435 | 19.75 | 6 | 90 | - | 11.64±1.25^b^ | 12.04±0.56^b^ | 13.03±0.98^b^ | 13.98±0.88^b^ | 12.98±0.79^b^ | 25.76±1.53^a^ |
| 15 | 5910-85-0 | 2,4-Heptadienal | 1500 | 1501 | 27.15 | 1 | 86 | - | 9.06±2.52^b^ | 10.69±0.85^ab^ | 17.22±2.26^a^ | 12.82±0.82^ab^ | 12.02±0.77^ab^ | 12.59±1.19^ab^ |
| 16 | 18829-56-6 | (*E*)-2-Nonenal | 1549 | 1543 | 28.36 | 6 | 95 | - | 4.69±0.72^b^ | 5.13±0.53^b^ | 5.3±0.17^b^ | 5.04±0.08^b^ | 5.99±0.27^b^ | 11.12±1.25^a^ |
| 17 | 557-48-2 | (*E*,*Z*)-2,6-Nonadienal | 1601 | 1593 | 29.98 | 8 | 96 | - | 1±0.22^a^ | 0.93±0.09^a^ | 1.6±0.34^a^ | 1.13±0.1^a^ | 1.61±0.09^a^ | 1.16±0.13^a^ |
| 18 | 30361-28-5 | (*E*,*E*)-2,4-Octadienal | 1607 | 1605 | 30.17 | 2 | 92 | - | 8.78±1.03^a^ | 5.37±0.51^ab^ | 8.4±1.62^a^ | - | 10.21±4.19^a^ | - |
| 19 | 2497-25-8 | (*Z*)-2-Decenal | 1658 | 1650 | 31.68 | 8 | 96 | - | 1.2±0.39^c^ | 0.89±0.13^bc^ | 1.2±0.02^bc^ | 1.33±0.14^bc^ | 2.5±0.59^b^ | 4.84±0.31^a^ |
| 20 | 25152-84-5 | (*E*,*E*)-2,4-Decadienal | 1829 | 1827 | 36.57 | 2 | 97 | - | 8.85±1.05^b^ | 7.48±0.4^b^ | 10.41±2.53^b^ | 10.59±1.82^b^ | 13.39±1.29^b^ | 21.28±1.25^a^ |
| 21 | 1576-87-0 | (*E*)-2-Pentenal | 1101 | 1117 | 13.15 | 16 | 93 | - | - | - | 4.48±0.75^a^ | - | - | - |
| 22 | 25152-83-4 | (*E*,*Z*)-2,4-Decadienal | 1780 | 1779 | 35.25 | 1 | 85 | - | - | - | - | 1.4±0.1^a^ | 1.53±0.19^a^ | 0.9±0.41^a^ |
| 23 | 6750-03-4 | 2,4-Nonadienal | 1677 | 1668 | 32.3 | 9 | 91 | - | - | - | - | - | - | 1.62±0.4^a^ |
| 24 | 67-64-1 | Acetone | 805 | 805 | 5.87 | 0 | 100 | 180.95±51.14^a^ | 203.5±29.73^a^ | 184.28±29.61^a^ | 192.1±41.03^a^ | - | - | - |
| 25 | 106-35-4 | 3-Heptanone | 1662 | 1661 | 15.32 | 1 | 96 | 25.82±7.9^a^ | 33.66±8.82^a^ | 23.57±7.52^ab^ | - | - | - | - |
| 26 | 110-43-0 | 2-Heptanone | 1187 | 1184 | 16.21 | 3 | 86 | - | 4.66±1.08^c^ | 8.52±0.25^bc^ | 17.49±4.18^bc^ | 38.66±8.56^a^ | 23.23±3.05^ab^ | 24.68±3.48^ab^ |
| 27 | 106-68-3 | 3-Octanone | 1259 | 1257 | 18.58 | 2 | 97 | 0.79±0.22^d^ | 15.01±0.77^a^ | 10.66±0.57^b^ | 9.23±1.39^b^ | 4.29±0.37^c^ | 3.55±0.08^c^ | 4.01±0.19^c^ |
| 28 | 111-13-7 | 2-Octanone | 1291 | 1290 | 19.74 | 1 | 88 | 1.48±0.35^b^ | 9.27±3.7^a^ | 12.01±1.24^a^ | 14.24±0.9^a^ | 13.63±0.52^a^ | 15.77±1.71^a^ | 14.54±0.76^a^ |
| 29 | 513-86-0 | Acetoin | 1300 | 1289 | 20.2 | 11 | 87 | 1.73±0.09^a^ | - | - | - | - | - | - |
| 30 | 110-93-0 | 6-Methyl-5-hepten-2-one | 1345 | 1341 | 21.67 | 4 | 96 | 0.82±0.29^b^ | 1.19±0.38^b^ | 1.12±0.31^b^ | 1.23±0.25^b^ | 1.85±0.08^ab^ | 2.9±0.31^a^ | 3.25±0.57^a^ |
| 31 | 821-55-6 | 2-Nonanone | 1396 | 1393 | 23.38 | 3 | 91 | 3.49±0.55^e^ | 26.38±1.88^d^ | 35.63±5.77^cd^ | 43.83±2.32^bc^ | 41.12±0.77^bc^ | 52.26±3.98^ab^ | 57.63±1.81^a^ |
| 32 | 98-86-2 | Acetophenone | 1673 | 1661 | 32.14 | 12 | 97 | 1.16±0.25^b^ | 2.44±0.73^ab^ | 3.62±0.37^ab^ | 6.81±2.96^ab^ | 4.67±0.45^ab^ | 4.87±0.34^ab^ | 7.66±0.57^a^ |
| 33 | 3796-70-1 | (*E*)-Geranylacetone | 1866 | 1860 | 37.58 | 6 | 94 | 0.7±0.17^d^ | 1.31±0.24^cd^ | 1.74±0.27^bcd^ | 2.37±0.41^bc^ | 2.64±0.16^b^ | 2.76±0.26^b^ | 4.44±0.22^a^ |
| 34 | 78-93-3 | 2-Butanone | 898 | 902 | 7.34 | 4 | 91 | - | 21.41±1.36^a^ | - | 20.05±3.38^a^ | - | - | - |
| 35 | 928-68-7 | 6-Methyl-2-heptanone | 1242 | 1237 | 18.03 | 5 | 99 | - | 9.1±0.8^b^ | 13.32±2.54^b^ | 13.71±1.68^b^ | 12.42±3.3^b^ | 12.79±0.92^b^ | 21.37±1.45^a^ |
| 36 | 4312-99-6 | 1-Octen-3-one | 1308 | 1305 | 20.35 | 3 | 91 | - | 12.84±1.26^b^ | 14.69±0.57^ab^ | 15.28±0.54^ab^ | 16.11±0.88^ab^ | 14.23±1.7^ab^ | 19.13±1.97^a^ |
| 37 | 8409-17-1 | (*E*)-3-Octen-2-one | 1418 | 1412 | 24.16 | 6 | 97 | - | 2.5±0.03^bc^ | - | 6.09±0.73^b^ | 5.59±0.25^b^ | 11.35±1.03^a^ | 14.5±2.3^a^ |
| 38 | 14309-57-0 | 3-Nonen-2-one | 1523 | 1508 | 27.57 | 15 | 91 | - | 3.41±0.37^a^ | 2.1±0.16^b^ | - | 1.52±0.1^b^ | 1.44±0.14^b^ | - |
| 39 | 600-14-6 | 2,3-Pentanedione | 1062 | 1062 | 11.86 | 0 | 86 | - | - | - | 1.23±0.19^a^ | - | - | - |
| 40 | 693-54-9 | 2-Decanone | 1502 | 1497 | 26.95 | 5 | 97 | - | - | 17.84±1.45^b^ | - | - | 48.02±3.84^a^ | - |
| 41 | 112-12-9 | 2-Undecanone | 1608 | 1602 | 30.22 | 6 | 86 | - | - | - | - | 1.19±0.05^b^ | - | - |
| 42 | 30086-02-3 | (*E*,*E*)-3,5-Octadien-2-one | 1553 | 1569 | 28.25 | 16 | 94 | - | - | - | - | - | - | 4.7±1.33^a^ |
| 43 | 123-51-3 | Isoamyl alcohol | 1215 | 1211 | 17.33 | 4 | 90 | 0.81±0.16^a^ | - | - | - | - | - | - |
| 44 | 626-89-1 | 4-Methyl-1-pentanol | 1319 | 1317 | 20.75 | 2 | 98 | 1.59±0.15^a^ | - | - | - | - | - | - |
| 45 | 111-27-3 | 1-Hexanol | 1358 | 1353 | 22.16 | 5 | 98 | 0.78±0.12^d^ | 2.49±0.36^cd^ | 3.31±0.2^c^ | 4.17±0.29^bc^ | 4.31±0.19^bc^ | 6.3±0.41^ab^ | 7.92±1.15^a^ |
| 46 | 3391-86-4 | 1-Octen-3-ol | 1454 | 1451 | 25.34 | 3 | 95 | 7.14±0.38^c^ | 285.03±78.85^b^ | 358.2±39.38^b^ | 457.1±58.06^ab^ | 446.56±23.33^ab^ | 468.12±28.81^ab^ | 585.77±45.95^a^ |
| 47 | 111-70-6 | 1-Heptanol | 1459 | 1456 | 25.53 | 3 | 93 | 0.03±0.01^e^ | 4.83±0.61^de^ | 5.67±0.32^de^ | 9.3±1.62^cd^ | 13.86±3.67^c^ | 22.58±1.64^b^ | 32.65±1.77^a^ |
| 48 | 104-76-7 | 2-Ethylhexanol | 1493 | 1490 | 26.63 | 3 | 99 | 2.27±0.42^e^ | 6.2±0.47^d^ | 7.44±1.61^cd^ | 10.09±0.46^bc^ | 10.32±0.24^bc^ | 11.9±0.58^ab^ | 13.92±0.37^a^ |
| 49 | 100-51-6 | Benzyl alcohol | 1893 | 1884 | 38.31 | 9 | 90 | 0.08±0^c^ | 1.08±0.28^b^ | 1.07±0.14^b^ | 1.26±0.1^b^ | 1.59±0.03^b^ | 1.4±0.1^b^ | 3.06±0.2^a^ |
| 50 | 616-25-1 | 1-Penten-3-ol | 1167 | 1160 | 15.32 | 7 | 86 | - | 5.16±1.15^ab^ | 11.3±3.26^a^ | - | - | 11.3±1.83^a^ | 11.17±2.35^a^ |
| 51 | 71-41-0 | 1-Pentanol | 1255 | 1250 | 18.74 | 5 | 89 | - | 13.6±3^c^ | 20.04±3.77^bc^ | 23.61±3.19^bc^ | 22.86±1.44^bc^ | 29.16±2.09^b^ | 41.73±1.95^a^ |
| 52 | 4730-22-7 | 6-Methyl-2-heptanol | 1376 | 1375 | 22.79 | 1 | 96 | - | 8.25±0.84^a^ | 7.91±0.41^a^ | 6.55±1.21^a^ | 1.7±0.06^b^ | - | - |
| 53 | 111-87-5 | 1-Octanol | 1562 | 1558 | 28.78 | 4 | 95 | - | 7.76±1.32^cd^ | 13.09±3.06^c^ | 13.28±2.54^c^ | 15.74±0.75^c^ | 31.06±2.63^b^ | 52.57±2.03^a^ |
| 54 | 18409-17-1 | (*E*)-2-Octen-1-ol | 1621 | 1614 | 30.6 | 7 | 99 | - | 27.17±1.17^d^ | 27.2±0.72^d^ | 32.52±3.74^cd^ | 38.09±1.06^bc^ | 44.55±2.16^b^ | 66.88±0.25^a^ |
| 55 | 60-12-8 | Phenethyl alcohol | 1927 | 1920 | 39.2 | 7 | 97 | - | - | - | - | 6.32±0.12^a^ | 5.4±0.39^a^ | 6.12±0.84^a^ |
| 56 | 143-08-8 | 1-Nonanol | 1661 | 1661 | 31.86 | 0 | 92 | - | - | - | - | - | - | 1.28±0.46^a^ |
| 57 | 123-66-0 | Ethyl hexanoate | 1229 | 1229 | 17.64 | 0 | 90 | 0.4±0.03^b^ | 0.89±0.04^a^ | - | 0.63±0.21^ab^ | - | - | - |
| 58 | 104-50-7 | gamma-Octalactone | 1728 | 1715 | 33.78 | 13 | 90 | - | 3.81±0.52^a^ | - | - | - | - | - |
| 59 | 706-14-9 | gamma-Caprolactone | 1726 | 1715 | 33.72 | 11 | 90 | - | - | - | - | 1.14±0.05^a^ | - | - |
| 60 | 64-19-7 | Acetic acid | 1469 | 1461 | 25.87 | 8 | 92 | 11.77±2.54^a^ | 16.03±3.24^a^ | 6.43±0.79^ab^ | 11.04±3.99^a^ | - | - | - |
| 61 | 503-74-2 | Isovaleric acid | 1682 | 1676 | 32.43 | 6 | 87 | 0.88±0.08^a^ | - | - | - | - | - | - |
| 62 | 142-62-1 | Capronic acid | 1857 | 1851 | 37.34 | 6 | 89 | 1.77±0.21^cd^ | 7.87±0.62^a^ | 3.76±0.27^bc^ | 6.07±1.46^ab^ | - | - | - |
| 63 | 124-07-2 | Caprylic acid | 2070 | 2062 | 42.82 | 8 | 95 | 6.08±1.82^a^ | 3.31±1.61^ab^ | 1.46±0.37^b^ | 0.57±0.07^b^ | - | - | - |
| 64 | 112-05-0 | Nonanoic acid | 2180 | 2176 | 45.36 | 4 | 81 | 0.62±0.07^b^ | 0.94±0.08^a^ | - | 0.53±0.11^b^ | - | - | - |
| 65 | 106-42-3 | p-Xylene | 1125 | 1135 | 14.15 | 10 | 95 | 12.33±4.22^a^ | 14.59±0.48^a^ | 16.27±0.67^a^ | 10.81±1.71^a^ | - | - | - |
| 66 | 95-47-6 | o-Xylene | 1186 | 1186 | 14..36 | 0 | 89 | 8.2±1.71^a^ | - | - | 11.33±1.89^a^ | - | - | - |
| 67 | 100-42-5 | Styrene | 1266 | 1261 | 18.74 | 5 | 99 | 4.83±1.36^c^ | 8±0.19^b^ | 9.19±0.76^b^ | 12.54±0.4^a^ | 14.09±0.25^a^ | 14.01±0.84^a^ | 15.3±0.4^a^ |
| 68 | 99-87-6 | p-Cymene | 1271 | 1272 | 18.96 | 1 | 99 | - | 0.73±0.05^a^ | - | - | - | - | - |
| 69 | 95-63-6 | 1,2,4-Trimethylbenzene | 1287 | 1284 | 19.51 | 3 | 81 | 1.33±0.4^b^ | 4.45±0.35^a^ | 2.09±0.22^b^ | - | - | - | - |
| 70 | 108-95-2 | Phenol | 2024 | 2015 | 41 | 9 | 99 | 1.42±0.09^c^ | 2.91±0.14^a^ | 2.07±0.21^bc^ | 2.62±0.56^b^ | 1.4±0.05^c^ | 1.12±0.11^c^ | - |
| 71 | 106-44-5 | p-Cresol | 2100 | 2090 | 43.57 | 10 | 93 | 1.99±0.06^bc^ | 4.27±0.29^a^ | 2.75±0.27^b^ | 2.76±0.57^b^ | 1.37±0.05^c^ | 0.97±0.06^cd^ | - |
| 72 | 620-17-7 | 3-Ethylphenol | 2193 | 2192 | 45.77 | 1 | 81 | 0.44±0.02^b^ | - | - | 0.87±0.17^a^ | - | - | - |
| 73 | 95-48-7 | o-Cresol | 2020 | 2012 | 41.57 | 8 | 90 | - | 0.84±0.36^cd^ | 2.06±0.08^bc^ | 2.24±0.56^bc^ | 2.89±0.22^ab^ | 2.57±0.41^ab^ | 3.78±0.32^a^ |
| 74 | 3777-69-3 | 2-Pentylfuran | 1229 | 1234 | 17.4 | 5 | 93 | 2.3±0.43^d^ | 7.77±0.79^d^ | 14.08±1.59^c^ | 20.89±2.27^b^ | 25.96±1.51^b^ | 24.89±1.68^b^ | 43.72±1.27^a^ |
| 75 | 98-01-1 | Furfural | 1480 | 1473 | 26.21 | 7 | 85 | 0.3±0.04^a^ | - | - | - | - | - | - |
| 76 | 123-32-0 | 2,5-Dimethylpyrazine | 1334 | 1324 | 21.27 | 10 | 98 | 4.72±0.5^a^ | - | - | - | - | - | - |
| 77 | 108-50-9 | 2,6-Dimethylpyrazine | 1340 | 1331 | 21.66 | 9 | 97 | 2.87±0.42^c^ | 8.51±0.58^c^ | 16.4±2.97^b^ | 21.69±1.97^b^ | 20.65±0.27^b^ | 29.46±2.01^a^ | 34.31±0.86^a^ |
| 78 | 14667-55-1 | 2,3,5-Trimethylpyrazine | 1415 | 1405 | 25.06 | 10 | 95 | 0.35±0.03^a^ | - | - | - | - | - | - |
| 79 | 109-08-0 | 2-Methylpyrazine | 1278 | 1267 | 19.46 | 11 | 86 | - | 5.29±0.78^b^ | 9.31±1.08^c^ | 11.61±0.94^c^ | 12.45±0.17^bc^ | 15.37±0.99^b^ | 24.28±0.94^a^ |
| 80 | 13925-00-3 | 2-Ethylpyrazine | 1344 | 1336 | 21.64 | 8 | 81 | - | - | - | - | - | 2.3±0.1^a^ | - |
| 81 | 13360-64-0 | 2-Ethyl-5-methylpyrazine | 1394 | 1387 | 23.36 | 7 | 86 | - | - | - | - | 5.23±0.2^c^ | 10.5±0.73^a^ | 7.13±0.14^b^ |
| 82 | 109-97-7 | Pyrrole | 1530 | 1518 | 27.79 | 12 | 96 | 1.79±0.06^b^ | 6.45±0.45^a^ | 5.48±0.73^a^ | 6.05±1.13^a^ | - | - | - |
| 83 | 120-72-9 | Indole | 2480 | 2463 | 52.05 | 17 | 95 | 7.29±0.24^c^ | 11.88±1.97^c^ | 9.49±0.82^c^ | 13.36±3.75^bc^ | 10.26±0.63^c^ | 19.56±1.83^ab^ | 25±0.52^a^ |
| 84 | 110-86-1 | Pyridine | 1196 | 1181 | 16.63 | 15 | 99 | - | 24.34±3.58^a^ | 19.77±2.57^a^ | 23.87±4.99^a^ | - | - | - |
| 85 | 551-93-9 | o-Aminoacetophenone | 2249 | 2233 | 47.02 | 16 | 90 | - | - | 0.68±0.06^b^ | 1.6±0.39^a^ | - | - | 1.95±0.4^a^ |
| 86 | 105-57-7 | Acetal | 881 | 892 | 7.073 | 11 | 90 | - | 23±1.43^a^ | 18.5±1.29^a^ | 18.85±3.64^a^ | - | - | - |

^a^: Retention indices (RI) calculated based on n-alkanes (C7-C34). ^b^: Retention indices (RI) were referred to NIST database(<https://webbook.nist.gov/chemistry/>). RT, retention time (min). SI, similarity index. Compounds were identified by NIST library matching (similarity ≥ 80%), retention indices (RI) comparison with literature values (ΔRI ≤ 20). ‘-’, no data found.

**Table S6**

Odor activity values (OAV) of volatile compounds in preserved egg yolk during pickling.

| No. | CAS | Compounds | OT(ng/g)^a^ | OAV | | | | | | |
| --- | --- | --- | --- | --- | --- | --- | --- | --- | --- | --- |
|  |  |  |  | 0d | 6d | 12d | 18d | 24d | 30d | 36d |
| 1 | 66-25-1 | Hexanal | 0.32 | 16 | 343 | 4＜17 | 464 | 49＜1 | 6＜15 | 962 |
| 2 | 124-19-6 | Nonanal | 0.32 | 7 | 57 | 82 | 112 | 126 | 232 | 442 |
| 3 | 112-31-2 | Decanal | 0.08 | 5 | 37 | - | 39 | - | - | - |
| 4 | 100-52-7 | Benzaldehyde | 24 | ＜1 | ＜1 | ＜1 | 1 | 2 | 1 | 2 |
| 5 | 122-78-1 | Benzeneacetaldehyde | 0.3 | 14 | - | - | - | - | - | - |
| 6 | 629-80-1 | Hexadecanal | - | - | - | - | - | - | - | - |
| 7 | 110-62-3 | Valeraldehyde | 2.7 | - | 4 | 5 | 5 | 6 | - | - |
| 8 | 497-03-0 | (*E*)-2-Methyl-2-butenal | 458.9 | - | ＜1 | - | - | - | - | - |
| 9 | 111-71-7 | Heptanal | 0.25 | - | 62 | 5＜1 | 55 | 47 | 58 | 85 |
| 10 | 107-86-8 | 3-Methyl-2-butenal | - | - | - | - | - | - | - | - |
| 11 | 6728-26-3 | (*E*)-2-Hexenal | 10 | - | ＜1 | ＜1 | ＜1 | - | - | ＜1 |
| 12 | 124-13-0 | Octanal | 0.32 | - | 23 | 37 | 38 | 4＜1 | 64 | 93 |
| 13 | 18829-55-5 | (*E*)-2-Heptenal | 13 | - | ＜1 | ＜1 | 1 | 1 | 1 | 1 |
| 14 | 2548-87-0 | (*E*)-2-Octenal | 0.34 | - | 34 | 35 | 38 | 41 | 38 | 75 |
| 15 | 5910-85-0 | 2,4-Heptadienal | 150 | - | ＜1 | ＜1 | ＜1 | ＜1 | ＜1 | ＜1 |
| 16 | 18829-56-6 | (*E*)-2-Nonenal | 0.08 | - | 58 | 64 | 66 | 62 | 74 | 138 |
| 17 | 557-48-2 | (*E*,*Z*)-2,6-Nonadienal | 0.0018 | - | 554 | 517 | 887 | 626 | 892 | 643 |
| 18 | 30361-28-5 | (*E*,*E*)-2,4-Octadienal | 10 | - | ＜1 | ＜1 | ＜1 | - | 1 | - |
| 19 | 2497-25-8 | (*Z*)-2-Decenal | 50 | - | ＜1 | ＜1 | ＜1 | ＜1 | ＜1 | ＜1 |
| 20 | 25152-84-5 | (*E*,*E*)-2,4-Decadienal | 0.027 | - | 327 | 277 | 385 | 392 | 495 | 788 |
| 21 | 1576-87-0 | (*E*)-2-Pentenal | 310 | - | - | - | ＜1 | - | - | - |
| 22 | 25152-83-4 | (*E*,*Z*)-2,4-Decadienal | 0.04 | - | - | - | - | 35 | 38 | 22 |
| 23 | 6750-03-4 | 2,4-Nonadienal | 0.05 | - | - | - | - | - | - | 32 |
| 24 | 67-64-1 | Acetone | 40 | 4 | 5 | 4 | 4 | - | - | - |
| 25 | 106-35-4 | 3-Heptanone | 7.5 | 3 | 4 | 3 | - | - | - | - |
| 26 | 110-43-0 | 2-Heptanone | 1 | - | 4 | 8 | 17 | 38 | 23 | 24 |
| 27 | 106-68-3 | 3-Octanone | 21 | ＜1 | ＜1 | ＜1 | ＜1 | ＜1 | ＜1 | ＜1 |
| 28 | 111-13-7 | 2-Octanone | 5 | ＜1 | 1 | 2 | 2 | 2 | 3 | 2 |
| 29 | 513-86-0 | Acetoin | 14 | ＜1 | ＜1 | ＜1 | ＜1 | ＜1 | ＜1 | ＜1 |
| 30 | 110-93-0 | 6-Methyl-5-hepten-2-one | 50 | ＜1 | ＜1 | ＜1 | ＜1 | ＜1 | ＜1 | ＜1 |
| 31 | 821-55-6 | 2-Nonanone | 5 | ＜1 | 5 | 7 | 8 | 8 | 1＜1 | 11 |
| 32 | 98-86-2 | Acetophenone | 36 | ＜1 | ＜1 | ＜1 | ＜1 | ＜1 | ＜1 | ＜1 |
| 33 | 3796-70-1 | (*E*)-Geranylacetone | 60 | ＜1 | ＜1 | ＜1 | ＜1 | ＜1 | ＜1 | ＜1 |
| 34 | 78-93-3 | 2-Butanone | 7000 | - | ＜1 | - | ＜1 | - | - | - |
| 35 | 928-68-7 | 6-Methyl-2-heptanone | 8.1 | - | 1 | 1 | 1 | 1 | 1 | 2 |
| 36 | 4312-99-6 | 1-Octen-3-one | 0.003 | - | 428＜1 | 4897 | 5＜194 | 5369 | 4742 | 6375 |
| 37 | 8409-17-1 | (*E*)-3-Octen-2-one | 20 | - | ＜1 | ＜1 | ＜1 | ＜1 | ＜1 | ＜1 |
| 38 | 14309-57-0 | 3-Nonen-2-one | 800 | - | ＜1 | ＜1 | - | ＜1 | ＜1 | - |
| 39 | 600-14-6 | 2,3-Pentanedione | 20 | - | - | - | ＜1 | - | - | - |
| 40 | 693-54-9 | 2-Decanone | 3 | - | - | 5 | - | - | 16 | - |
| 41 | 112-12-9 | 2-Undecanone | 5.5 | - | - | - | - | ＜1 | - | - |
| 42 | 30086-02-3 | (*E*,*E*)-3,5-Octadien-2-one | 100 | - | - | - | - | - | - | ＜1 |
| 43 | 123-51-3 | Isoamyl alcohol | 4 | ＜1 | - | - | - | - | - | - |
| 44 | 626-89-1 | 4-Methyl-1-pentanol | 820 | ＜1 | - | - | - | - | - | - |
| 45 | 111-27-3 | 1-Hexanol | 5.6 | ＜1 | ＜1 | ＜1 | ＜1 | ＜1 | 1 | 1 |
| 46 | 3391-86-4 | 1-Octen-3-ol | 0.005 | 1428 | 57＜1＜16 | 71639 | 9142＜1 | 89312 | 93623 | 117153 |
| 47 | 111-70-6 | 1-Heptanol | 5.4 | ＜1 | ＜1 | 1 | 1 | 2 | 4 | 6 |
| 48 | 104-76-7 | 2-Ethylhexanol | 198 | ＜1 | ＜1 | ＜1 | ＜1 | ＜1 | ＜1 | ＜1 |
| 49 | 100-51-6 | Benzyl alcohol | 100 | ＜1 | ＜1 | ＜1 | ＜1 | ＜1 | ＜1 | ＜1 |
| 50 | 616-25-1 | 1-Penten-3-ol | 358.1 | - | ＜1 | ＜1 | - | - | ＜1 | ＜1 |
| 51 | 71-41-0 | 1-Pentanol | 120 | - | ＜1 | ＜1 | ＜1 | ＜1 | ＜1 | ＜1 |
| 52 | 4730-22-7 | 6-Methyl-2-heptanol | 39 | - | ＜1 | ＜1 | ＜1 | ＜1 | - | - |
| 53 | 111-87-5 | 1-Octanol | 42 | - | ＜1 | ＜1 | ＜1 | ＜1 | ＜1 | 1 |
| 54 | 18409-17-1 | (*E*)-2-Octen-1-ol | 20 | - | 1 | 1 | 1 | 1 | 2 | 3 |
| 55 | 60-12-8 | Phenethyl alcohol | 0.015 | - | - | - | - | 421 | 36＜1 | 4＜17 |
| 56 | 143-08-8 | 1-Nonanol | 34 | - | - | - | - | - | - | - |
| 57 | 123-66-0 | Ethyl hexanoate | 0.01 | 4＜1 | 89 | - | 63 | - | - | - |
| 58 | 104-50-7 | gamma-Octalactone | 6.5 | - | ＜1 | - | ＜1 | - | - | - |
| 59 | 706-14-9 | gamma-Caprolactone | 0.7 | - | - | - | - | 1 | - | - |
| 60 | 64-19-7 | Acetic acid | 22000 | ＜1 | ＜1 | ＜1 | ＜1 | - | - | - |
| 61 | 503-74-2 | Isovaleric acid | 12 | ＜1 | - | - | - | - | - | - |
| 62 | 142-62-1 | Capronic acid | 35.6 | ＜1 | ＜1 | ＜1 | ＜1 | - | - | - |
| 63 | 124-07-2 | Caprylic acid | 910 | ＜1 | ＜1 | ＜1 | ＜1 | - | - | - |
| 64 | 112-05-0 | Nonanoic acid | 3000 | ＜1 | ＜1 | - | ＜1 | - | - | - |
| 65 | 106-42-3 | p-Xylene | 530 | ＜1 | ＜1 | ＜1 | ＜1 | - | - | - |
| 66 | 95-47-6 | o-Xylene | 450.23 | ＜1 | - | - | ＜1 | - | - | - |
| 67 | 100-42-5 | Styrene | 3.6 | 1 | 2 | 2 | 3 | 3 | 3 | 4 |
| 68 | 99-87-6 | p-Cymene | 5.01 | - | ＜1 | - | - | - | - | - |
| 69 | 95-63-6 | 1,2,4-Trimethylbenzene | 260 | ＜1 | ＜1 | ＜1 | - | - | - | - |
| 70 | 108-95-2 | Phenol | 31 | ＜1 | ＜1 | ＜1 | ＜1 | ＜1 | ＜1 | - |
| 71 | 106-44-5 | p-Cresol | 8 | ＜1 | ＜1 | ＜1 | ＜1 | ＜1 | ＜1 | - |
| 72 | 620-17-7 | 3-Ethylphenol | 0.85 | ＜1 | - | - | 1 | - | - | - |
| 73 | 95-48-7 | o-Cresol | 260 | - | ＜1 | ＜1 | ＜1 | ＜1 | ＜1 | ＜1 |
| 74 | 3777-69-3 | 2-Pentylfuran | 5.8 | ＜1 | 1 | 2 | 3 | 4 | 4 | 7 |
| 75 | 98-01-1 | Furfural | 282 | ＜1 | - | - | - | - | - | - |
| 76 | 123-32-0 | 2,5-Dimethylpyrazine | 80 | ＜1 | - | - | - | - | - | - |
| 77 | 108-50-9 | 2,6-Dimethylpyrazine | 400 | ＜1 | ＜1 | ＜1 | ＜1 | ＜1 | ＜1 | ＜1 |
| 78 | 14667-55-1 | 2,3,5-Trimethylpyrazine | 23 | ＜1 | - | - | - | - | - | - |
| 79 | 109-08-0 | 2-Methylpyrazine | 60 | - | ＜1 | ＜1 | ＜1 | ＜1 | ＜1 | ＜1 |
| 80 | 13925-00-3 | 2-Ethylpyrazine | 4000 | - | - | - | - | - | ＜1 | - |
| 81 | 13360-64-0 | 2-Ethyl-5-methylpyrazine | 16 | - | - | - | - | ＜1 | ＜1 | ＜1 |
| 82 | 109-97-7 | Pyrrole | 10000 | ＜1 | ＜1 | ＜1 | ＜1 | - | - | - |
| 83 | 120-72-9 | Indole | 11 | ＜1 | 1 | ＜1 | 1 | ＜1 | 1 | 2 |
| 84 | 110-86-1 | Pyridine | 0.03 | - | 811 | 658 | 795 | - | - | - |
| 85 | 551-93-9 | o-Aminoacetophenone | 0.2 | - | - | 3 | 8 | - | - | 9 |
| 86 | 105-57-7 | Acetal | 4.9 | - | 4 | 3 | 3 | - | - | - |

^a^: Odor thresholds were referenced as a published book, named: Odor thresholds-compilations of odor threshold values in air, water and other media (2nd ed). ‘-’, no data found.

Note: Volatile quantification data for OAV calculation were determined using GC-MS

**Table S7**

Identification of volatile compounds from preserved egg white during pickling by GC-IMS.

| No. | Compounds | Formula | MW | RI | Rt (s) | Dt |
| --- | --- | --- | --- | --- | --- | --- |
| 1 | 1-Penten-3-one-M | C5H8O | 84.1 | 700.2 | 194.942 | 1.06103 |
| 2 | 2-Ethylfuran-M | C6H8O | 96.1 | 725.6 | 214.73 | 1.04428 |
| 3 | 2-Butanone-M | C4H8O | 72.1 | 587.1 | 141.538 | 1.05709 |
| 4 | Propanal | C3H6O | 58.1 | 511.7 | 115.303 | 1.04418 |
| 5 | 2-Propanol | C3H8O | 60.1 | 520.1 | 117.971 | 1.07688 |
| 6 | 2-Propanone | C3H6O | 58.1 | 497.2 | 110.857 | 1.11992 |
| 7 | 2-Methylpropanal | C4H8O | 72.1 | 582.5 | 139.759 | 1.09754 |
| 8 | 1-Propanol | C3H8O | 60.1 | 555.8 | 129.977 | 1.12939 |
| 9 | 2-Butanol | C4H10O | 74.1 | 590.2 | 142.737 | 1.15007 |
| 10 | 2,3-Butandione | C4H6O2 | 86.1 | 572.8 | 136.147 | 1.17521 |
| 11 | Methyl acetate | C3H6O2 | 74.1 | 517 | 116.977 | 1.17738 |
| 12 | Ethyl formate | C3H6O2 | 74.1 | 517.7 | 117.222 | 1.23015 |
| 13 | Propanoic acid | C3H6O2 | 74.1 | 690.2 | 187.619 | 1.10059 |
| 14 | Isopropyl acetate | C5H10O2 | 102.1 | 675.5 | 179.957 | 1.15523 |
| 15 | 1-Butanol | C4H10O | 74.1 | 656.9 | 171.084 | 1.18064 |
| 16 | Isobutyl formate | C5H10O2 | 102.1 | 695.4 | 191.37 | 1.19727 |
| 17 | 2-Butanone-D | C4H8O | 72.1 | 583.5 | 140.158 | 1.24561 |
| 18 | 2-Ethylfuran-D | C6H8O | 96.1 | 689 | 186.742 | 1.2965 |
| 19 | 1-Penten-3-one-D | C5H8O | 84.1 | 693.7 | 190.136 | 1.3266 |
| 20 | Pentanal | C5H10O | 86.1 | 692.7 | 189.446 | 1.42014 |
| 21 | Butanal | C4H8O | 72.1 | 606.1 | 149.033 | 1.28454 |
| 22 | 2-Methylpropanal | C4H8O | 72.1 | 588.6 | 142.095 | 1.2871 |
| 23 | 2-Hexanone | C6H12O | 100.2 | 781 | 265.248 | 1.19008 |
| 24 | Hexanal | C6H12O | 100.2 | 795.9 | 280.389 | 1.26379 |
| 25 | 3-Methyl-2-butenal | C5H8O | 84.1 | 788.8 | 273.085 | 1.36077 |
| 26 | 2-Methylpyrazine | C5H6N2 | 94.1 | 789.1 | 273.433 | 1.39414 |
| 27 | 3-Pentanone | C5H10O | 86.1 | 731 | 219.176 | 1.35132 |
| 28 | Benzaldehyde-M | C7H6O | 106.1 | 961 | 517.6 | 1.14808 |
| 29 | 1-Octen-3-ol | C8H16O | 128.2 | 984.7 | 565.391 | 1.1568 |
| 30 | (Z)-4-Heptenal | C7H12O | 112.2 | 886 | 391.548 | 1.15085 |
| 31 | 3-Heptanone | C7H14O | 114.2 | 865.2 | 362.547 | 1.22751 |
| 32 | 2-Heptanone | C7H14O | 114.2 | 885.4 | 390.731 | 1.25817 |
| 33 | 5-Methyl-3-heptanone-M | C8H16O | 128.2 | 936 | 471.608 | 1.27521 |
| 34 | Heptanal-M | C7H14O | 114.2 | 895.6 | 405.844 | 1.33398 |
| 35 | 2-Phenylacetaldehyde | C8H8O | 120.2 | 1026.7 | 642.878 | 1.26026 |
| 36 | 2-Pentylfuran | C9H14O | 138.2 | 993.2 | 583.52 | 1.25002 |
| 37 | Isobutyl butyrate | C8H16O2 | 144.2 | 952.3 | 501.207 | 1.34132 |
| 38 | 2-ethyl-1-hexanol | C8H18O | 130.2 | 1041.8 | 670.636 | 1.40773 |
| 39 | Octanal-M | C8H16O | 128.2 | 1011.7 | 616.446 | 1.40669 |
| 40 | 3-Octanol | C8H18O | 130.2 | 987.1 | 570.487 | 1.41603 |
| 41 | Benzaldehyde-D | C7H6O | 106.1 | 958.8 | 513.445 | 1.46379 |
| 42 | Styrene | C8H8 | 104.2 | 886.4 | 392.141 | 1.43055 |
| 43 | Nonanal | C9H18O | 142.2 | 1102.9 | 795.478 | 1.47205 |
| 44 | 2,5-Dimethylpyrazine | C6H8N2 | 108.1 | 887.3 | 393.513 | 1.49903 |
| 45 | 2-Ethyl-5-methylpyrazine | C7H10N2 | 122.2 | 1010.9 | 615.074 | 1.66504 |
| 46 | Benzyl alcohol | C7H8O | 108.1 | 1041.5 | 669.95 | 1.78332 |
| 47 | Octanal-D | C8H16O | 128.2 | 1012.1 | 617.132 | 1.81237 |
| 48 | Heptanal-D | C7H14O | 114.2 | 895.7 | 405.86 | 1.68683 |
| 49 | n-Hexanol | C6H14O | 102.2 | 865.7 | 363.134 | 1.32635 |
| 50 | Heptanol | C7H16O | 116.2 | 933.1 | 466.559 | 1.39839 |
| 51 | 3-Hexanone | C6H12O | 100.2 | 772.8 | 257.041 | 1.17046 |
| 52 | Ethyl butyrate | C6H12O2 | 116.2 | 792.4 | 276.76 | 1.55254 |
| 53 | Ethyl acetate | C4H8O2 | 88.1 | 654.5 | 169.987 | 1.31996 |
| 54 | 3-Methylbutanal | C5H10O | 86.1 | 660.4 | 172.751 | 1.40135 |
| 55 | 1-Pentanol | C5H12O | 88.1 | 759.9 | 244.716 | 1.25467 |
| 56 | 5-Methyl-3-heptanone-D | C8H16O | 128.2 | 932.1 | 464.851 | 1.68371 |

MW: Molecular Weight, RI: Retention Index, Rt: Retention time, Dt: Drift time. Monomers are represented by the symbol of “M”. Dimers are represented by the symbol of “D”.

The databases used for matching included NIST 2020 retention index databases (DB-5 and HP-5), Hanon 2024 MXT-5, CUSTOM Siloxanes SE54, and the following general mobility libraries: GAS 3H_IMS, GAS 3H_IMS_Silox, Hanon 2024-POS/NEG, Breath-Compounds, and Foodpairing-beeraroma.

**Table S8**

Identification of volatile compounds from preserved egg yolk during pickling by GC-IMS.

| No. | Compounds | Formula | MW | RI | Rt (s) | Dt |
| --- | --- | --- | --- | --- | --- | --- |
| 1 | Nonanal | C9H18O | 142.2 | 1104.2 | 798.54 | 1.47249 |
| 2 | (E)-2-Octenal-M | C8H14O | 126.2 | 1064.6 | 714.805 | 1.33066 |
| 3 | (E,E)-2,4-Heptadienal | C7H10O | 110.2 | 1021.2 | 633.058 | 1.19106 |
| 4 | 1-Penten-3-ol | C5H10O | 86.1 | 675.9 | 180.165 | 0.93501 |
| 5 | Furan | C4H4O | 68.1 | 556.7 | 130.319 | 0.91392 |
| 6 | 3-Penten-2-one | C5H8O | 84.1 | 722 | 211.812 | 1.06375 |
| 7 | Thiophene | C4H4S | 84.1 | 665.1 | 174.934 | 1.06166 |
| 8 | 2-Butanone-M | C4H8O | 72.1 | 589.6 | 142.499 | 1.06096 |
| 9 | Propanal | C3H6O | 58.1 | 507.7 | 114.063 | 1.05294 |
| 10 | 3-Methyl-2-butenal | C5H8O | 84.1 | 776.9 | 261.131 | 1.092 |
| 11 | (E)-2-Pentenal-M | C5H8O | 84.1 | 747.7 | 233.584 | 1.1056 |
| 12 | 3-Pentanone | C5H10O | 86.1 | 692.3 | 189.152 | 1.11083 |
| 13 | Butanal-M | C4H8O | 72.1 | 594.4 | 144.376 | 1.09712 |
| 14 | 2-Methylpropanal | C4H8O | 72.1 | 523.9 | 119.202 | 1.09664 |
| 15 | Acetone-M | C3H6O | 58.1 | 516.4 | 116.792 | 1.11842 |
| 16 | Acetoxyacetone | C5H8O3 | 116.1 | 492.8 | 109.553 | 1.09881 |
| 17 | 2-Propanethiol | C3H8S | 76.2 | 554.5 | 129.548 | 1.12805 |
| 18 | Acetic acid | C2H4O2 | 60.1 | 588.8 | 142.185 | 1.14602 |
| 19 | 2-Pentanol | C5H12O | 88.1 | 703.5 | 197.404 | 1.19386 |
| 20 | Ethyl formate | C3H6O2 | 74.1 | 638.4 | 162.689 | 1.22395 |
| 21 | 2-Butanone-D | C4H8O | 72.1 | 584.3 | 140.457 | 1.24391 |
| 22 | 3-Methylbutanal | C5H10O | 86.1 | 658.3 | 171.743 | 1.17608 |
| 23 | Methyl acetate | C3H6O2 | 74.1 | 557 | 130.431 | 1.19381 |
| 24 | Acetone-D | C3H8O | 60.1 | 517.8 | 117.24 | 1.23941 |
| 25 | 3-Methylbutan-1-ol | C5H12O | 88.1 | 728.7 | 217.287 | 1.24479 |
| 26 | Propanoic acid | C3H6O2 | 74.1 | 691.3 | 188.418 | 1.26506 |
| 27 | Pyrazine | C4H4N2 | 80.1 | 708.4 | 201.11 | 1.27234 |
| 28 | Hexanal | C6H12O | 100.2 | 814.7 | 300.598 | 1.26017 |
| 29 | 1-Pentanol | C5H12O | 88.1 | 760.7 | 245.427 | 1.2546 |
| 30 | 2-Methylpentanal | C6H12O | 100.2 | 760.7 | 245.427 | 1.51068 |
| 31 | 2-Heptanone | C7H14O | 114.2 | 884.7 | 389.72 | 1.26119 |
| 32 | Heptanal | C7H14O | 114.2 | 896.2 | 406.696 | 1.33117 |
| 33 | n-Hexanol | C6H14O | 102.2 | 865.1 | 362.37 | 1.32508 |
| 34 | 2-Methylpyrazine | C5H6N2 | 94.1 | 791.3 | 275.606 | 1.39303 |
| 35 | Cyclopentanone | C5H8O | 84.1 | 792.7 | 277.021 | 1.33725 |
| 36 | (E)-2-Hexenal-D | C6H10O | 98.1 | 845.1 | 336.435 | 1.5122 |
| 37 | Ethyl butanoate | C6H12O2 | 116.2 | 801.7 | 286.451 | 1.55378 |
| 38 | 2-Ethylfuran | C6H8O | 96.1 | 692.5 | 189.313 | 1.32559 |
| 39 | 1-Penten-3-one | C5H8O | 84.1 | 654.8 | 170.1 | 1.31693 |
| 40 | Pentanal | C5H10O | 86.1 | 696.4 | 192.111 | 1.41079 |
| 41 | 1-Butanol | C4H10O | 74.1 | 654.3 | 169.888 | 1.40125 |
| 42 | 1-Octen-3-ol | C8H16O | 128.2 | 984.9 | 565.814 | 1.15663 |
| 43 | Benzaldehyde-M | C7H6O | 106.1 | 960.4 | 516.481 | 1.14939 |
| 44 | 2-Phenylacetaldehyde | C8H8O | 120.2 | 1038.5 | 664.48 | 1.26039 |
| 45 | 2-Pentylfuran | C9H14O | 138.2 | 993.3 | 583.697 | 1.25074 |
| 46 | 1-octen-3-one | C8H14O | 126.2 | 980.5 | 556.564 | 1.27004 |
| 47 | (E)-2-Heptenal-M | C7H12O | 112.2 | 955.9 | 507.848 | 1.25315 |
| 48 | 3-Methylvaleric acid | C6H12O2 | 116.2 | 933.4 | 467.148 | 1.27728 |
| 49 | 2-ethyl-1-Hexanol | C8H18O | 130.2 | 1042.2 | 671.264 | 1.4112 |
| 50 | Hexyl acetate | C8H16O2 | 144.2 | 1011 | 615.147 | 1.40396 |
| 51 | 3-Octanol | C8H18O | 130.2 | 987.8 | 571.981 | 1.41724 |
| 52 | Butyl butanoate | C8H16O2 | 144.2 | 989 | 574.448 | 1.33881 |
| 53 | 1-Heptanol | C7H16O | 116.2 | 976.6 | 548.548 | 1.39552 |
| 54 | Isobutyl butyrate | C8H16O2 | 144.2 | 955.2 | 506.615 | 1.33519 |
| 55 | 2-Octanol | C8H18O | 130.2 | 989 | 574.448 | 1.46308 |
| 56 | Benzaldehyde-D | C7H6O | 106.1 | 960.4 | 516.481 | 1.4655 |
| 57 | 2-ethyl-5-Methylpyrazine | C7H10N2 | 122.2 | 1009.9 | 613.297 | 1.67301 |
| 58 | 2,4,5-Trimethylthiazole | C6H9NS | 127.2 | 984.6 | 565.198 | 1.59338 |
| 59 | 5-methyl-2-Furanmethanol | C6H8O2 | 112.1 | 958.8 | 513.398 | 1.56443 |
| 60 | (Z)-3-Hexenyl acetate | C8H14O2 | 142.2 | 1010.6 | 614.531 | 1.81659 |
| 61 | (E)-2-Heptenal-D | C7H12O | 112.2 | 956.2 | 508.465 | 1.65974 |
| 62 | 3-Hexanone | C6H12O | 100.2 | 807 | 292.187 | 1.4684 |
| 63 | 2,6-Dimethylpyridine | C7H9N | 107.2 | 903 | 417.127 | 1.08737 |
| 64 | (Z)-4-Heptenal | C7H12O | 112.2 | 888.2 | 394.835 | 1.15058 |
| 65 | (E)-2-Hexenal-M | C6H10O | 98.1 | 845.3 | 336.772 | 1.18159 |
| 66 | 2-Furanmethanethiol | C5H6OS | 114.2 | 919.8 | 444.086 | 1.33841 |
| 67 | Styrene | C8H8 | 104.2 | 895.9 | 406.241 | 1.41593 |
| 68 | 2-Ethylpyridine | C7H9N | 107.2 | 895.6 | 405.722 | 1.47019 |
| 69 | 3-Methylthiopropanal | C4H8OS | 104.2 | 934.2 | 468.51 | 1.4039 |
| 70 | Propyl butanoate | C7H14O2 | 130.2 | 895.6 | 405.817 | 1.68826 |
| 71 | 4-methyl-1-Pentanol | C6H14O | 102.2 | 897.4 | 408.47 | 1.62513 |
| 72 | Methyl hexanoate | C7H14O2 | 130.2 | 933.3 | 466.831 | 1.68452 |
| 73 | 3-Octanone | C8H16O | 128.2 | 986.7 | 569.627 | 1.71822 |
| 74 | Butanal-D | C4H8O | 72.1 | 591.2 | 143.133 | 1.28874 |
| 75 | Dimethyl sulfide | C2H6S | 62.1 | 482.9 | 106.628 | 0.92794 |
| 76 | (E)-2-Pentenal-D | C5H8O | 84.1 | 746 | 232.133 | 1.35488 |
| 77 | (E)-2-Octenal-D | C8H14O | 126.2 | 1066.6 | 718.766 | 1.80603 |

MW: Molecular Weight, RI: Retention Index, Rt: Retention time, Dt: Drift time. Monomers are represented by the symbol of “M”. Dimers are represented by the symbol of “D”.

The databases used for matching included NIST 2020 retention index databases (DB-5 and HP-5), Hanon 2024 MXT-5, CUSTOM Siloxanes SE54, and the following general mobility libraries: GAS 3H_IMS, GAS 3H_IMS_Silox, Hanon 2024-POS/NEG, Breath-Compounds, and Foodpairing-beeraroma.

**Fig. S1.** Permutation tests (200 times) for the PLS-DA models of volatile compounds (GC-MS) in egg white (A) and egg yolk (B), and for the PLS-DA models of non-volatile compounds (LC-MS) in egg white (C) and egg yolk (D).
